# Distinguishing two distinct types of salivary extracellular vesicles: a potential tool for understanding their pathophysiological roles

**DOI:** 10.3389/fmolb.2024.1278955

**Published:** 2024-02-28

**Authors:** Yuko Ogawa, Yuri Miura, Mamoru Ikemoto, Atsushi Ohnishi, Yoshikuni Goto, Kazuma Aoki, Yuki Motokurumada, Yoshihiro Akimoto, Tamao Endo, Masafumi Tsujimoto, Ryohei Yanoshita

**Affiliations:** ^1^ Faculty of Pharmaceutical Sciences, Teikyo Heisei University, Tokyo, Japan; ^2^ Research Team for Mechanism of Aging, Tokyo Metropolitan Institute of Gerontology, Tokyo, Japan; ^3^ Department of Anatomy, Kyorin University School of Medicine, Tokyo, Japan

**Keywords:** extracellular vesicles (EVs), exosomes, microvesicles, human whole saliva, dipeptidyl peptidase IV (DPP IV), aminopeptidase N (APN), size-exclusion chromatography, differential centrifugation

## Abstract

Extracellular vesicles (EVs), which are found in almost all cells and human body fluids, are currently being studied as a source of pathophysiological information. Previously, we demonstrated that at least two types of EVs can be isolated from human whole saliva (WS) using enzymatic activity of dipeptidyl peptidase IV (DPP IV) as a marker for differentiating the EV subsets. In the present study, EV fractions, termed EV-I 20 k-ppt and EV-II 100 k-ppt, were prepared by a combination of size-exclusion chromatography of improved condition and sequential centrifugation. The EV-I 20 k-ppt fraction contained medium/large EVs with a diameter of 100–1,000 nm, including aminopeptidase N (APN), mucin 1, ezrin, and Annexin A1. EV-II 100 k-ppt contained small EVs with a diameter of 20–70 nm, with DPP IV and CD9, programmed cell death 6-interacting protein, and tumor susceptibility gene 101 as characteristic proteins. Proteomic analyses also revealed distinctive repertoires of constituent proteins. Immunoprecipitation of several membrane proteins of the EVs with respective antibodies suggested their differential local membrane environment between the two types of salivary vesicles. Thus, we identified two distinctive types of EVs, one is APN/MUC1- rich EVs (EV-I, large/medium EVs) and the other is DPP IV/CD9-rich EVs (EV-II, small EVs). Furthermore, analysis of the binding of the EVs to coronavirus spike proteins showed that EV-II 100 k-ppt, but not EV-I 20 k-ppt, significantly bound to the spike protein of Middle East respiratory syndrome coronavirus (MERS-CoV). Finally, we developed a simple method to prepare two distinctive EVs from only 1 mL of human WS using sequential immunoprecipitation. Elucidating the features and functions of these two types of salivary EVs may help us understand their pathophysiological roles in the oral cavity and gastrointestinal tract.

## Introduction

Extracellular vesicles (EVs) have attracted attention as novel participants in intercellular communication. EVs deliver contents with important pathophysiological functions. They are produced and released by diverse cellular lineages such as reticulocytes, lymphocytes, dendritic cells, and intestinal epithelial cells. They can also be detected in various body fluids, such as blood, breast milk, malignant ascites, urine, amniotic fluid, and saliva. They contain various RNAs and proteins, which are released into the extracellular environment in response to various stimuli. It has been shown that vesicular mRNA and microRNA (miRNA) transferred to other cells can function under the novel environmental conditions there (Valadi et al., 2007). During the metastatic process, tumor-derived exosomal integrins selectively adhere to their target organ where they can promote the formation of pre-metastatic niches (Hoshino et al., 2015). The levels of miR-4484 in salivary exosomes have been reported to be significantly increased among individuals with oral lichen planus, a persistent inflammatory disease affecting the oral mucosa (Byun et al., 2015). In addition, Yu et al. demonstrated that coagulant tissue factor and CD24 of salivary EVs bind to activated platelets and trigger coagulation (Yu et al., 2018). These findings reveal that EV-associated proteins and miRNA play important pathophysiological roles by interacting with their target cells.

EVs with diameters of 30–100 nm are referred to as small EVs (sEVs) or exosomes. Other types of EVs are designated as medium/large EVs (m/lEVs) or microvesicles. sEVs/exosomes often contain tetraspanins, including CD9, CD63, and CD81, and cytosolic proteins such as programmed cell death 6-interacting protein (Alix), tumor susceptibility gene 101 (TSG101), and syntenin-1 (Jeppesen et al., 2019; Kugeratski et al., 2021). Regarding m/lEVs, Jeppesen et al. showed that Annexin A1 is a specific marker for large EVs (lEVs)/microvesicles shed directly from the plasma membrane (Jeppesen et al., 2019). Additionally, ezrin, from the ezrin/radixin/moesin family that links the plasma membrane to the actin cytoskeleton, is transported along with microvesicular cargo, and thus is considered a marker of microvesicles (Bian et al., 2019). These results imply that EVs derived from the same sources may be heterogeneous and exert various functions depending on the pathophysiological and/or environmental conditions. A single cell line has been shown to secrete EVs of different sizes and contents, confirming the heterogeneity of EVs (Jeppesen et al., 2019). The Minimal Information for Studies of Extracellular Vesicles 2018 (MISEV 2018) report stated that no consensus has yet been reached on specific markers of EV subtypes; therefore, assigning an EV to a specific biogenesis pathway remains extremely difficult (Théry et al., 2018).

Human whole saliva (WS) is a mixture of aqueous proteins and minerals. It is derived from the major and minor salivary glands, as well as from gingival crevicular fluid. WS plays a crucial role as the first line of defense through its lubricating, antiviral, antibacterial, and buffering properties. Owing to the ease, relatively low cost, and noninvasiveness of collection, WS has been used for health and disease monitoring in human subjects. Our research has demonstrated that WS contains abundant EVs, and dipeptidyl peptidase IV (DPP IV/CD26) in saliva was shown to be predominantly associated with the membrane of salivary EVs (Ogawa et al., 2008). We also separated EVs based on their size and found at least two EV types. One was sEVs/exosomes, classified by the expression of several proteins, such as CD9, Alix, and TSG101 (Ogawa et al., 2011). Using these EVs, we performed proteomic and transcriptomic analyses that led to the identification of many proteins and RNAs, some of which may play crucial roles in the pathophysiological and defensive functions of the oral cavity (Ogawa et al., 2013; Ogawa et al., 2016). In addition, we examined the morphological stability and membrane integrity of salivary EVs, revealing their remarkable stability, with them retaining their membrane integrity for a prolonged period when stored at 4°C (Kumeda et al., 2017; Ogawa et al., 2021). However, the procedures for separating the salivary EV subpopulations that we employed were still inadequate and the pathophysiological roles of these subpopulations remain incompletely understood.

In the present study, we found that the enzymatic activity of aminopeptidase N (APN) was identifiable in salivary EVs and could be a marker of them, along with DPP IV activity. In addition to these two activities, we found high levels of mucin 1 (MUC1) expression on the surface of salivary m/lEVs/microvesicles. Using these three proteins (APN, DPP IV, and MUC1) as markers, we improved the procedures to separate the two subpopulations of EVs more distinctively than ever before. Significant differences were found between the two types of salivary EVs in the contents and abilities to bind particular antibodies or viral spike proteins, suggesting functional diversity. Our results should facilitate elucidation of the pathophysiological significance of salivary EVs.

## Materials and methods

### Study ethical approval and sample collection

Ethical approval for this study was obtained from the institutional review board of Teikyo Heisei University (approval number R01-109-2), Tokyo Metropolitan Institute of Gerontology and Geriatric Hospital (approval number R23-01), and Kyorin University School of Medicine (approval number H29-146-0l). This study was conducted according to the principles of the Declaration of Helsinki. Human WS samples were collected from 11 healthy volunteers, aged 22–50 years (donors A–K), at our laboratory (Supplementary Table S1). Written informed consent was obtained from all volunteers before sample collection. Donors were negative for severe acute respiratory syndrome coronavirus 2 (SARS-CoV-2). The donors were asked to refrain eating, drinking, smoking, or oral hygiene procedures for at least 2 h prior to sample collection. At 9–12 a.m., the donors were directed to spit 40 mL of unstimulated WS into a 50 mL tube. The sample was placed 25°C upon collection and then immediately used.

### Isolation of EVs from human whole saliva

The EVs were purified from the WS in accordance with a previously described method (Ogawa et al., 2011), with minor modifications. Briefly, 40 mL of WS was subjected to centrifugation at 6,000 × *g* for 15 min at 20°C (himac CF16RXII, T9A31 rotor; Koki Holdings Co., Ltd., Tokyo, Japan) to remove cell debris and bacterial pellets. The supernatant was then filtered through a 5.0-µm cellulose acetate filter (17594-K; Sartorius AG, Göttingen, Germany) and concentrated to an approximate volume of 1 mL using an Amicon Ultra-15 centrifugal filter device that had an exclusion cut-off of 100 kDa (Millipore Corporation, Bedford, MA, USA). Subsequently, the concentrated filtrate (1.5–2.0 mL) was purified using size-exclusion chromatography on a Sephacryl S-1000 SF (GE Healthcare UK Ltd. Little Chalfont, UK) column (1.5 cm × 50 cm, 0.33 mL/min) equilibrated with Tris-buffered saline (20 mM Tris-HCl, pH 7.4, and 150 mM NaCl), and 80 fractions (1.2 mL/fraction) were collected (FC 204 Fraction Collector; Gilson, Middleton, WI, USA) within 4 h. All fractions were analyzed for DPP IV and APN activity (see below, “Aminopeptidase activity”), and absorbance at 280 nm. Fractions that corresponded to a small peak of absorbance with high APN [fraction (Fr.) EV-I, fraction number 22-31] or DPP IV (Fr. EV-II, fraction number 36-49) activity were pooled and subsequently filtered using a 0.45-µm cellulose acetate filter (2062-025; AGC TECHNO GLASS Co., Ltd., Shizuoka, Japan). The pooled fractions were concentrated and exchanged with phosphate-buffered saline (PBS, pH 7.4, 2.7 mM KCl, 1.5 mM KH_2_PO_4_, 136.9 mM NaCl, and 8.9 mM Na_2_HPO_4_·7H_2_O) using the Amicon Ultra-4 with a 100 kDa cut-off. The protein concentration of the fractions was measured using a Pierce™ BCA Protein Assay Kit (23235; Thermo Fisher Scientific, Waltham, MA, USA), in accordance with the manufacturer’s instructions. The concentrated fractions were used for subsequent characterization studies. Isolated EV fractions were stored at 4°C for further studies up to 1 month. All relevant data from this study have been submitted to the EV-TRACK knowledgebase (EV-TRACK ID: EV230571) (EV-TRACK Consortium et al., 2017).

### Aminopeptidase activity

The DPP IV activity was estimated through a previously described assay (Ogawa et al., 2008). Briefly, assay mixtures containing 50 µL of 0.4 mM Gly-Pro-MCA, 100 µL of 100 mM Tris-HCl (pH 8.5), and 50 µL of enzyme solution were prepared. Highly concentrated samples were diluted to 50 µL. Therefore, 10 µL of each fraction obtained by size-exclusion chromatography was used. After 20-min incubation at 37°C, 2.8 mL of 1 M sodium acetate (pH 4.2) was added to the assay mixture to terminate the reaction. The fluorescence intensity, which corresponded to the released 7-amino-4-methyl-coumarin, was measured at 460 nm, with excitation at 380 nm (FP-6300; JASCO Corporation, Tokyo, Japan). APN activity was assayed using the same method as that used for DPP IV activity, with assay mixtures containing 50 µL of 0.4 mM Ala-MCA and 100 µL of 100 mM Tris-HCl (pH 7.4).

### Sodium dodecyl sulfate–polyacrylamide gel electrophoresis and western blotting

From the EV fractions prepared as described above, samples of 2 µg protein were separated using sodium dodecyl sulfate polyacrylamide gel electrophoresis (SDS-PAGE) on SuperSep HG 5%–20% gradient gels (FUJIFILM Wako Pure Chemical Corporation, Osaka, Japan). Silver staining (2D-Silver Stain Reagent II; Cosmo Bio, Tokyo, Japan) was performed to visualize the protein bands. To detect specific proteins in salivary EVs, the protein bands on the gel were transferred onto polyvinylidene difluoride (PVDF) membranes (BSP0161; Pall Corporation, Westborough, MA, USA) using the wet transfer method (1703930JA; Bio-Rad Laboratories, Inc., Hercules, CA, USA). The transfer was carried out at 30 V for 15 h. Nonspecific binding sites on the PVDF membranes were blocked by incubating the membranes in 100 mM Tris-HCl (pH 7.4) and 150 mM NaCl with 5% skim milk and 1% Tween 20. Subsequently, the membranes were incubated overnight at 4°C with primary antibodies, rabbit anti-mucin 5B (MUC5B, polyclonal antibody, 1:1,000, H-300; Santa Cruz Biotechnology, Inc., Dallas, TX, USA), goat anti-IgA (polyclonal antibody, 1:1,000, A80-102A; Bethyl Laboratories, Montgomery, TX, USA), mouse anti-MUC1 (monoclonal antibody, 1:1,000, NBP2-29408; Novus Biologicals, Littleton, CO, USA), goat anti-DPP IV (polyclonal antibody, 1:1,000; AF1180, R&D Systems, Inc., Minneapolis, MN, US), rabbit anti-CD9 (1:1,000, EXOAB-CD9A-1; System Biosciences, Mountain View, CA, USA), goat anti-Alix (polyclonal antibody, 1:1,000, Q-19; Santa Cruz Biotechnology, Inc.), mouse anti-TSG101 (monoclonal antibody, 1:1,000, 4A10; Abcam, Cambridge, MA, USA), mouse anti-APN (monoclonal antibody, 1:1,000, sc-166105; Santa Cruz Biotechnology, Inc.), rabbit anti-ezrin (polyclonal antibody, 1:1,000, #3145; Cell Signaling Technology, Danvers, MA, USA), or mouse anti-Annexin A1 (monoclonal antibody, 1:1,000, MAB37701; R&D Systems, Inc.). Following this, the membranes were further incubated at 25°C for 1 h with horseradish peroxidase (HRP)-labeled secondary antibodies [1:5,000; Rabbit anti-Goat IgG(H+L) HRP Conjugate, 81-1620, Thermo Fisher Scientific; Goat anti-Rabbit IgG (H+L), HRP Conjugate, W4011, or Goat anti-Mouse IgG(H+L) HRP Conjugate, W4021, Promega, Madison, WI, USA]. An LAS 4000 mini luminescent image analyzer (GE Healthcare Bio-Science Corp., Piscataway, NJ, USA) and ECL Prime Western Blotting Detection Kit (GE Healthcare) were used to visualize the protein bands. As positive controls (MUC1, APN, DPP IV, CD9, Alix, TSG101, ezrin, Annexin A1), we selected THP-1 and Caco-2 cell line from the cell line data on the Human Protein Atlas database (Karlsson et al., 2021). Cell lysates were prepared using RIPA buffer, and 5–20 μg of protein was applied to gels. In addition, we used WS (10 μg), IgA from human colostrum (1 μg, I1010, Sigma-Aldrich; St. Louis, MO, USA), recombinant APN (rAPN; 100 ng, 3815-ZN; R&D Systems, Inc.), rDPP IV (5 ng, 9168-SE; R&D Systems, Inc), and recombinant mouse Annexin A1 (PMA32995, Assaypro, Charles, MO, USA) were also used as positive controls.

### Sequential centrifugation

Fr. EV-I or Fr. EV-II proteins (100 μg) that had been separated by size-exclusion chromatography and exchanged with PBS by Amicon Ultra-4 ultrafiltration, were further subjected to sequential centrifugation at 20,000 × *g* for 30 min at 4°C (himac CS150NX, P100AT2 rotor; Koki Holdings Co., Ltd., Tokyo, Japan) to sediment m/lEVs/microvesicles (20 k-ppt). The resultant supernatant (sup) was then subjected to ultracentrifugation at 100,000 × *g* for 18 h at 4°C (himac CS150NX, P100AT2 rotor; Koki Holdings Co., Ltd.) to sediment the sEVs/exosomes (100 k-ppt). The recovered precipitate was washed, suspended in 50–100 μL of PBS, and used as a 20 k-ppt or 100 k-ppt fraction. The BCA Protein Assay Kit (23235; Thermo Fisher Scientific) was used to determine the protein concentration of each fraction, in accordance with the manufacturer’s instructions.

### Transmission electron microscopy

Transmission electron microscopy (TEM) analysis was performed in accordance with a previously described method (Ogawa et al., 2011), with a few modifications. In brief, the EV fractions, prepared as described earlier, were mixed with 25% glutaraldehyde (G011/1, TAAB Laboratories Equipment Ltd., Aldermaston, UK) in phosphate buffer (pH 7.2) at a ratio of 9:1. The 2.5 µL of samples were then applied onto collodion-coated 200-mesh copper grids (collodion support film on 200 mesh copper grids, No. 6511, Nisshin EM Corporation, Tokyo, Japan). After standing the samples for 5 s, the grids were then stained with 2% uranyl acetate (pH 7.0) for 5 s. Excess uranyl acetate solution on the grids was absorbed with filter paper and embedded in 2% methylcellulose/0.4% uranyl acetate (pH 4.0). The grids were then dried and examined using TEM (TEM-1010; JEOL, Tokyo, Japan) at 80 kV.

### Dynamic light scattering

The size distribution profile of salivary EVs was studied via dynamic light scattering (DLS) based on the laser diffraction method using a Zetasizer Nano ZS90 (Malvern Instruments, Malvern, UK). EV fractions possessing proteins at a concentration of 10 μg/mL were analyzed at a constant temperature (25°C); the buffer viscosity was 0.8872 cP, and the buffer refractive index, particle absorption, and particle refractive index were set to 1.33 η, 0.010, and 1.59 η, respectively. The DLS signal intensity was transformed to the volume distribution [volume (%)], assuming that the EVs were spherical in shape. The evaluation software provided by the supplier (Malvern Zetasizer Software 7.13) is based on the cumulant method and uses the Stokes–Einstein equation for size determination. Measurements were repeated at least three times for each sample (derived from each donor).

### Nano liquid chromatography tandem mass spectrometry (nanoLC-MS/MS)

EV-I 20 k-ppt or EV-II 100 k-ppt proteins (100–200 μg) were concentrated using a Vivacon 500 (125 kDa exclusion; Sartorius AG) and dissolved in 0.1% RapiGest SF (Waters, Milford, MA, USA) in 50 mM triethylammonium bicarbonate (TEAB) buffer (pH 8.5). The solubilized EV proteins (8 μg) were diluted with 40 μL of 50 mM TEAB buffer and subsequently reduced with 2.4 mM tris(2-carboxyethyl) phosphine hydrochloride (TCEP) (Sciex, Framingham, MA, USA) at 60°C for 1 h. Finally, the cysteine residues were blocked with 9 mM methyl methanethiosulfonate (MMTS; Sciex) at 25°C for 30 min in the dark. The proteins were then digested with 1 μg of Trypsin Gold (MS-grade; Promega) at 37°C for 18 h. The digests were acidified by the addition of trifluoroacetic acid (TFA) and incubated at 37°C for 30 min. This sample was then centrifuged at 17,000 × *g* for 10 min to remove the RapiGest SF. The supernatants were collected and concentrated *in vacuo*. The residues were dissolved in 100 μL of 2% acetonitrile (MeCN) containing 0.1% TFA and desalted using a MonoSpin C18 spin column (GL Sciences Inc., Tokyo, Japan). Eluates were evaporated *in vacuo* and dissolved in 20 μL of 0.1% formic acid (FA).

LC-MS/MS analyses were performed using an UltiMate 3000 RSLCnano System (Thermo Fisher Scientific) coupled to a Q Exactive hybrid quadrupole-Orbitrap mass spectrometer with a nano ESI source (Thermo Fisher Scientific), as described previously (Miura et al., 2023). Peptide separation was performed using a 90-min gradient of water containing 0.1% FA (mobile phase A) and MeCN containing 0.1% FA (mobile phase B) at a flow rate of 300 nL/min. The elution gradient was set as follows: 0–3  min, 2% B; 3–93  min, 2%–40% B; 93–95  min, 40%–95% B; 95–105  min, 95% B; 105–107  min, 95%–2% B; and 107–120  min, 2% B. Mass spectrometry was performed in data-dependent acquisition mode. The MS parameters were as follows: spray voltage, 2.0 kV; capillary temperature, 275°C; S-lens RF level, 50; scan type, full MS; scan range, *m/z* 350–1,500; resolution, 70,000; polarity, positive; automatic gain control target, 3 × 10^6^; and maximum injection time, 100 ms. The MS/MS parameters were as follows: resolution, 17,500; automatic gain control target, 1 × 10^5^; maximum injection time, 60 ms; normalized collision energy (NCE), 27; dynamic exclusion, 15 s; loop count, 10; isolation window, 1.6 *m/z*; and charge exclusion: unassigned, 1 and ≥8. The injection volume was 2 µL (containing 0.8 µg proteins). For each sample, measurements were collected in duplicate.

### Protein identification and gene ontology (GO) analysis

Proteome Discoverer 2.4 software (Thermo Fisher Scientific) was used for protein identification and Gene Ontology (GO) analysis. The analytical parameters used for the database search were as follows: parent mass tolerance, 10.0 ppm; fragment mass error tolerance, 0.02 Da; false discovery rate (FDR) confidence, <0.01; search engine, sequest HT; protein database, Swissprot (*Homo sapiens*); enzyme name, trypsin (full); maximum numbers of missed cleavages, 2; static modification, MMTS (cysteine); and dynamic modifications, oxidation (methionine), phosphorylation (serine, threonine, and tyrosine), acetyl (lysine), acetyl (N-terminus), Met-loss (N-terminus methionine), Met-loss+acetyl (N-terminus methionine). Protein identification was considered to be correct if it fulfilled the following selection criteria: protein FDR confidence had at least a high level, and this high level was found in at least three samples. We performed GO analysis on selected protein lists from EV-I and EV-II sample groups. We included only those proteins that were confidently identified in either the EV-I or the EV-II sample group.

### Cleavage of peptide substrates by EVs

Peptide substrates (25 μM) were incubated with EV-I or EV-II (30 μg/mL) at 37°C for 60 min in PBS. The reaction was terminated by the addition of TFA and MeCN [the final concentration was adjusted to the high-performance liquid chromatography (HPLC) mobile phase]. The reaction products were separated on a reversed-phase column COSMOSIL 5C18-AR-300 (4.6 mm inner diameter × 250 mm; Nacalai Tesque, Kyoto, Japan), using an automated HPLC system (LC-2010AHT; Shimadzu, Kyoto, Japan). After incubation with the EVs, peptide fragments were isocratically eluted at a flow rate of 0.5 mL/min with 20% MeCN containing 0.086% TFA for kallidin or 25% MeCN containing 0.086% TFA for substance P. For confirmation of the effect of inhibitors on the aminopeptidase activity, salivary EVs (30 μg/mL) and 1 μM inhibitor [amastatin, (4095-v, Peptide Institute Inc., Osaka, Japan) or alogliptin (9656-50, BioVision Inc., Milpitas, CA, USA)] were mixed with PBS on ice for 5 min and then incubated with each peptide at 37°C for 60 min.

### Effect of inhibitors on aminopeptidase activity

For evaluation of the inhibitory effects of amastatin or alogliptin benzoate, we mixed Fr. EV-I or Fr. EV-II (0.5–2 μg/mL) with various concentrations of inhibitors in 100 μL of PBS on ice for 5 min. The samples were then incubated with 25 μM Ala-MCA for Fr. EV-I or Gly-Pro-MCA for Fr. EV-II at 37°C for 20 min, and the amount of 7-amino-4-methyl-coumarine released was measured as described earlier. Following incubation at 37°C for 20 min, the reaction was terminated by the addition of 2.8 mL of 1 M sodium acetate (pH 4.2). The fluorescence intensity corresponding to the released 7-amino-4-methyl-coumarin was measured at 460 nm, with excitation at 380 nm (FP-6300; JASCO Corporation).

### Enzyme-linked immunosorbent assay

Enzyme-linked immunosorbent assay (ELISA) plates were coated with 0.5 μg of spike proteins of Middle East respiratory syndrome coronavirus [MERS-CoV (40069-V08B1, Sino Biological Inc. Beijing, China) or SARS-CoV-2 (40589-V08B1, Sino Biological Inc.)] in 50 μL/well of 50 mM carbonate buffer (pH 9.6) overnight at 4°C. Plates were blocked with 150 μL of 3% bovine serum albumin (BSA, Sigma-Aldrich) in PBS for 1 h at 25°C. After blocking, plates were incubated with 0.5 μg of EV-I or EV-II in 1% BSA–PBS for 2 h at 25°C. Plates were then washed three times with Tris-buffered saline (TBS) with 0.05% Tween 20 (TTBS) and incubated with 50 μL of mouse anti-APN antibody (Santa Cruz Biotechnology, Inc.) for EV-I or goat anti-DPP IV antibody (R&D Systems, Inc.) for EV-II diluted 1:1,000 with 1% BSA–PBS overnight at 4°C. After washing, plates were incubated at 25°C for 1.5 h with 50 μL of Goat anti-Mouse IgG(H+L) HRP Conjugate (Promega) for anti-APN antibody diluted 1:5,000 with 1% BSA–PBS, or Rabbit anti-Goat IgG(H+L) HRP Conjugate (Thermo Fisher Scientific) for anti-DPP IV antibody diluted 1:10,000 with 1% BSA–PBS. After washing five times with TTBS and once with PBS, 100 μL of substrate mixture [0.4 mg/mL *o*-phenylenediamine (Sigma-Aldrich) and 0.015% H_2_O_2_ in citrate/phosphate buffer (pH 5.0)] was added to develop color for 30 min. The reaction was terminated with 50 μL of 2 M HCl and the absorbance at 492 nm was measured using a microplate reader. All experiments were performed in triplicate, with two repetitions.

### Immunoprecipitation

Salivary EVs (Fr. EV-I or Fr. EV-II) were immunoprecipitated using magnetic beads (epoxy-activated Dynabeads, Dynabeads Antibody Coupling Kit, 14311D; Thermo Fisher Scientific) conjugated with mouse anti-MUC1 (Novus Biologicals), goat anti-DPP IV (R&D Systems, Inc.), or sheep anti-APN (polyclonal antibody, AF3815; R&D Systems, Inc.), in accordance with the manufacturer’s instructions. Magnetic beads (5 mg) and antibodies (25 μg) were used in the coupling reaction. We also used Dynabeads Exosome Human CD9 Isolation Reagent (10614D; Thermo Fisher Scientific). For the control experiment, we prepared magnetic beads conjugated with nonspecific IgGs from mice [anti-MUC1 or anti-CD9; IgG from mouse serum (I5381) Sigma-Aldrich], goats [anti-DPP IV; IgG from goat serum (I5256) Sigma-Aldrich], and sheep [anti-APN; IgG from sheep serum (I6131) Sigma-Aldrich]. Antibody-coupled beads were added to 5 μg of EVs and the mixture was incubated at 4°C for 18 h on a rotator. After incubation, the beads were washed thrice with 1 mL of PBS. Then, the beads were added to 20 μL of sample buffer and incubated at 70°C for 10 min. The eluates were then subjected to western blotting (see above). The secondary antibodies used were Mouse TrueBlot ULTRA (eB144): anti-mouse IgG HRP, Goat TrueBlot (eB270): anti-goat IgG HRP, and Rabbit TrueBlot (eB182): anti-rabbit IgG HRP (Rockland Immunochemicals, Limerick, PA, USA), which do not bind denatured IgG. Anti-Sheep IgG, Donkey-Poly, HRP (HAF016; R&D Systems, Inc.), was used for an anti-APN antibody. Experiments were performed at least twice on three samples (derived from each donor).

### Sequential immunoprecipitation of salivary EVs

Two types of salivary EVs were sequentially immunoprecipitated from the WS using magnetic beads (epoxy-activated Dynabeads). WS (1.0 mL) was subjected to centrifugation at 6,000 × *g* for 15 min at 20°C and filtered through a 5.0-µm cellulose acetate filter (see Isolation of EVs from human WS). The beads were washed thrice with 1 mL of PBS. Then, the anti-DPP IV antibody-coupled beads were added to 1 mL of pretreated WS, and the mixture was incubated at 4°C for 3 h on a rotator (first immunoprecipitation). After separating the beads, WS was further immunoprecipitated using MUC1 antibody-coupled beads at 4°C for 18 h on a rotator (second immunoprecipitation). For the control experiment, nonspecific IgGs of goat (for first immunoprecipitation) or mouse (for second immunoprecipitation) were used. The immunoprecipitated beads were washed thrice with 1 mL of PBS. Then, the beads were added to 20 μL of sample buffer and incubated at 70°C for 10 min. The eluates were then subjected to western blotting.

### Statistical analysis

All data are representative of at least three independent experiments and are presented as the mean ± standard deviation (SD). Statistical analysis was performed using one-way analysis of variance (ANOVA), and *p* < 0.05 was considered to indicate statistical significance. GraphPad Prism 9.5.1 (GraphPad Software, La Jolla, CA, USA) software was used to perform statistical analyses.

## Results

### Identification and separation of EV-I and EV-II extracellular vesicles

**FIGURE 1:**
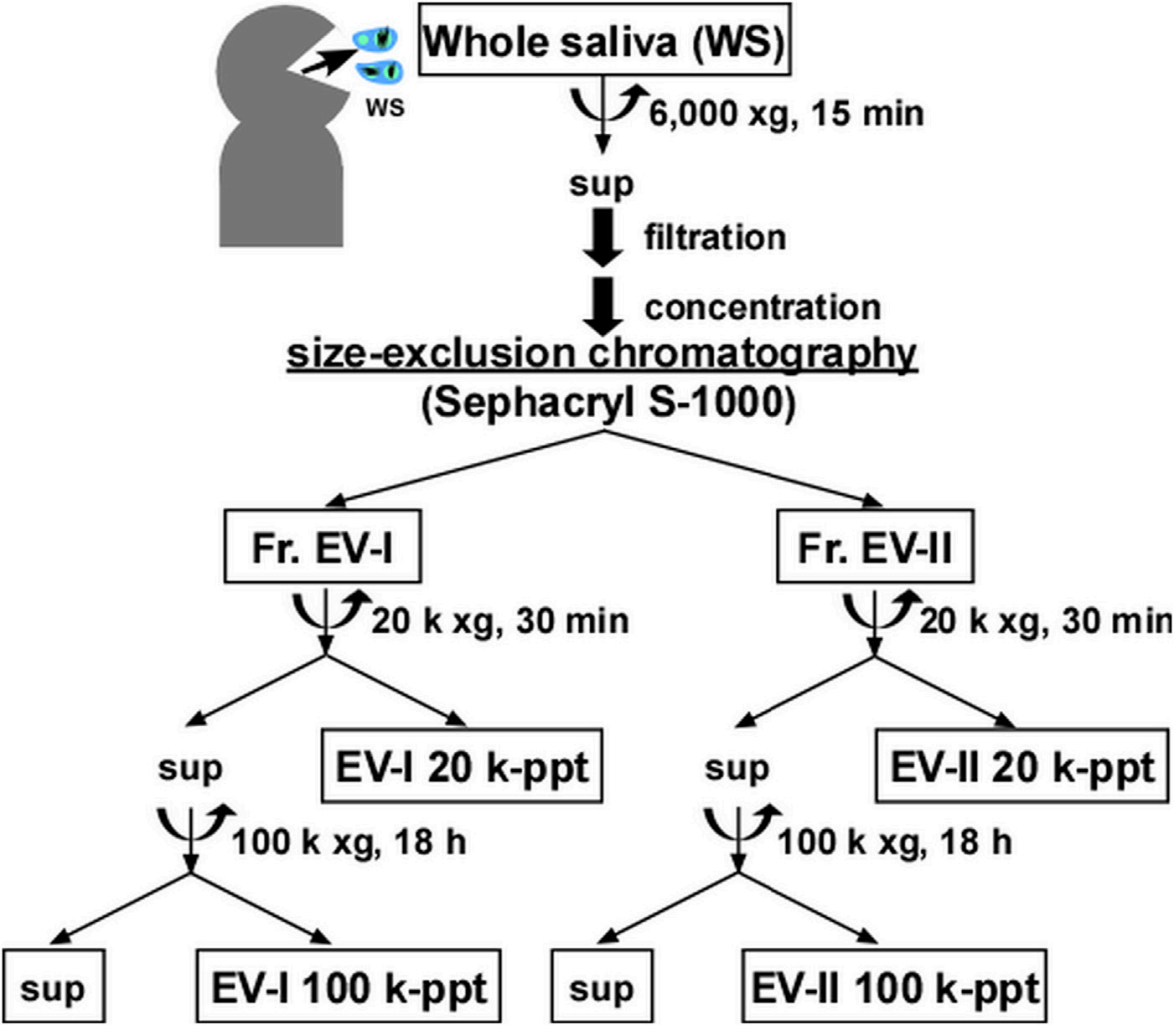
Flow chart of procedure for isolating salivary extracellular vesicles. Salivary extracellular vesicles (EVs) were isolated using a combination of size-exclusion chromatography and sequential centrifugation. Human whole saliva was centrifuged, and the supernatant was filtrated and then, concentrated using Amicon Ultra-15. The resulting concentrated solution was applied to a size-exclusion column, and the fractions (Fr.) of EV-I and Fr. EV-II were determined from the measurement of OD280, DPP IV activity, and APN activity. Each fraction was collected, filtered, and concentrated by Amicon Ultra-4 to obtain Fr. EV-I and Fr. EV-II. Each pooled fraction was subjected to sequential centrifugation. The precipitate obtained by centrifugation at 20,000 × *g* (EV-I or EV-II 20 k-ppt) and the precipitate obtained by centrifugation at 100,000 × *g* (EV-I or EV-II 100 k-ppt) were further analyzed. After characterizing the protein components (SDS-PAGE and western blotting) and morphological analyses (TEM and DLS), we focused on EV-I 20 k-ppt and EV-II 100 k-ppt and subjected them to proteomic analyses.

Figure 1 shows a schematic overview of the EV separation procedures. In a previous study (Ogawa et al., 2011), using DPP IV as a marker, we prepared human salivary EVs using a combination of ultrafiltration and size-exclusion column chromatography with a Sephacryl S-500 column and reported their characteristics. Although the EVs appeared to be eluted as a single peak, we divided them into two fractions and found subpopulations with different EV sizes. To further characterize salivary EVs, we performed size-exclusion column chromatography with Sephacryl S-1000 instead of S-500 to improve the separation efficiency. In addition, we found that APN activity was associated with a certain subset of salivary EVs during the initial course of this study and could be used as a marker for differentiating the EV subsets.

**FIGURE 2:**
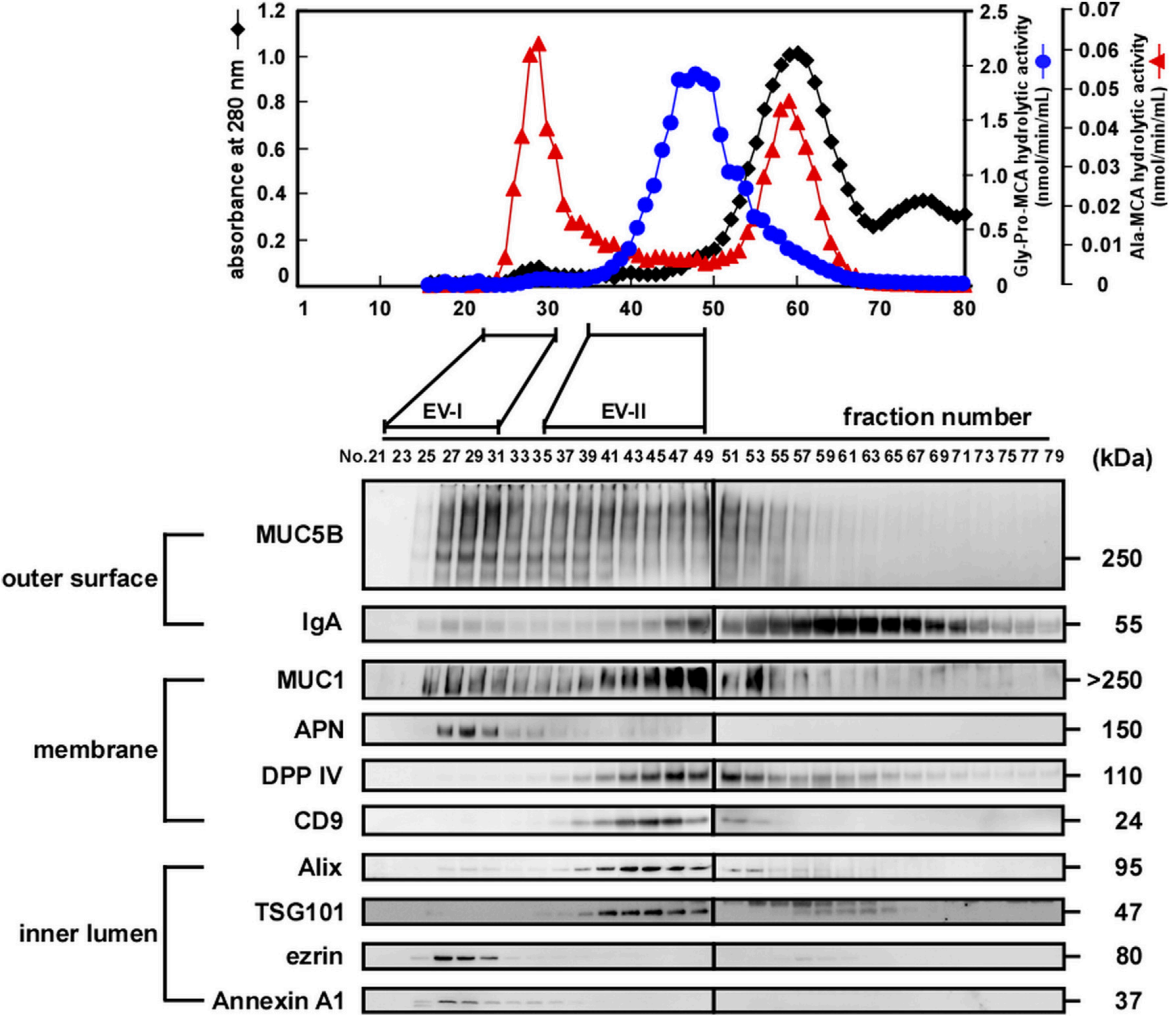
Preparation of EVs derived from human whole saliva. (upper) Size-exclusion chromatography (Sephacryl S-1000 SF) elution profiles of the EVs from fresh human WS (donor A). The filtrated and concentrated WS (1.5–2.0 mL) was subjected to size-exclusion chromatography on a Sephacryl S-1000 SF column (1.5 cm × 50 cm, 0.33 mL/min) equilibrated with Tris-buffered saline (20 mM Tris-HCl, pH 7.4, and 150 mM NaCl), and 80 fractions (1.2 mL/fraction) were collected within 4 h. All fractions were analyzed for DPP IV and APN activities, and absorbance at 280 nm. The experiments were performed at least three times for each donor, and a representative elution pattern is shown. (lower) Western blot analysis of proteins located on the outer surface (MUC5B and IgA), membrane (MUC1, APN, DPP IV, and CD9), and inner lumen (Alix, TSG101, ezrin, and Annexin A1) of the salivary EV fractions eluted from size-exclusion columns (donor A). Numbers refer to the different fractions obtained via size-exclusion column chromatography shown in the upper panel. Overall, 20 µL of each EV fraction was subjected to SDS-PAGE and analyzed using western blotting. The elution profiles and western blotting of EVs from other donors are shown in Supplementary Figure S1.

The upper panels of Figure 2 (donor A) and Supplementary Figure S1A (donor B and donor C) show elution profiles from the Sephacryl S-1000 column of WS prepared from a single volunteer by monitoring enzymatic activities of APN (shown by Ala-MCA degrading activity) and DPP IV (shown by Gly-Pro-MCA degrading activity). The APN and DPP IV activities were clearly separated. Then, fractions 36–49 were pooled and designated as EV-II fractions (Fr. EV-II). Meanwhile, APN activity eluted as two peaks, one of which eluted near the void volume and the other as molecules with fractions 55–65. Fractions 22–32 were pooled and designated as the EV-I fraction (Fr. EV-I). The results of APN and DPP IV activities in the isolation are summarized in Supplementary Table S2.

We then compared the protein components of Frs. EV-I and EV-II using western blot analysis of several proteins reported as being associated with salivary EVs (lower panels of Figure 2; Supplementary Figures S1A,B). Outer surface-associated proteins, MUC5B and IgA, were eluted rather diffusely, and some proportion of each protein appeared to co-elute with Fr. EV-I or EV-II. MUC5B binds to membrane-associated MUC1 on the oral epithelial cells (Ployon et al., 2016), and MUC5B and IgA form a complex on the oral mucosa (Gibbins et al., 2015). In addition, IgA and MUC5B were still detectable from the precipitate after sequential centrifugation (see below), suggesting their association with salivary EVs. It has been shown that a specific interaction of blood proteins with the surface of vesicles, known as a protein corona, also forms in EVs (Tóth et al., 2021). MUC1, which is known to be membrane-bound, was also co-eluted with Frs. EV-I and EV-II. Consistent with the APN enzymatic activity (Figure 2, upper panel), a large proportion of APN was detected in Fr. EV-I and fractions around 60. APN was detected as a 150 kDa band by western blotting in Fr. EV-I. In contrast, a band at 120 kDa is detected in the fraction around 60 (Supplementary Figure S1C). Low molecular weight APN may be soluble APN due to proteolytic cleavage, deglycosylation, or both, during APN processing. In addition, these fractions may contain some oral bacterial enzymes with Ala-MCA degrading activity (Suido et al., 1986). Co-elution of DPP IV and CD9, both of which are membrane-bound proteins, was apparent in Fr. EV-II, whereas their association with Fr. EV-I was marginal.

The elution patterns of cytosolic EV-associated proteins, such as Alix, TSG101, ezrin, and Annexin A1, of Fr. EV-I differed from those of EV-II. Alix and TSG101 were eluted from the column in Fr. EV-II. However, their co-elution with Fr. EV-I was barely detectable. In contrast, the elutions of ezrin and Annexin A1 in Fr. EV-I were apparent, whereas those in Fr. EV-II were marginal. Thus, plausibly, there are at least two distinct EV fractions that show different co-elution patterns of EV-associated proteins. Fr. EV-I is an APN-ezrin- and Annexin A1-rich and DPP IV-CD9-Alix- and TSG101-poor fraction. In contrast to Fr. EV-I, Fr. EV-II is an APN-ezrin- and Annexin A1-poor and DPP IV-CD9-Alix- and TSG101-rich fraction. Monitoring of APN and DPP IV activities resulted in clear separation of the two fractions; therefore, we decided to prepare the EV fractions using these two activities as new EV markers. Studies have reported that m/lEVs are precipitated by centrifugation at 20,000 × g (Kowal et al., 2016; Jeppesen et al., 2019), and therefore we were prompted to further characterize EV-I and EV-II by using sequential centrifugations. The centrifugation conditions were set as described in Figure 1 and the *Materials and Methods* section.

**FIGURE 3:**
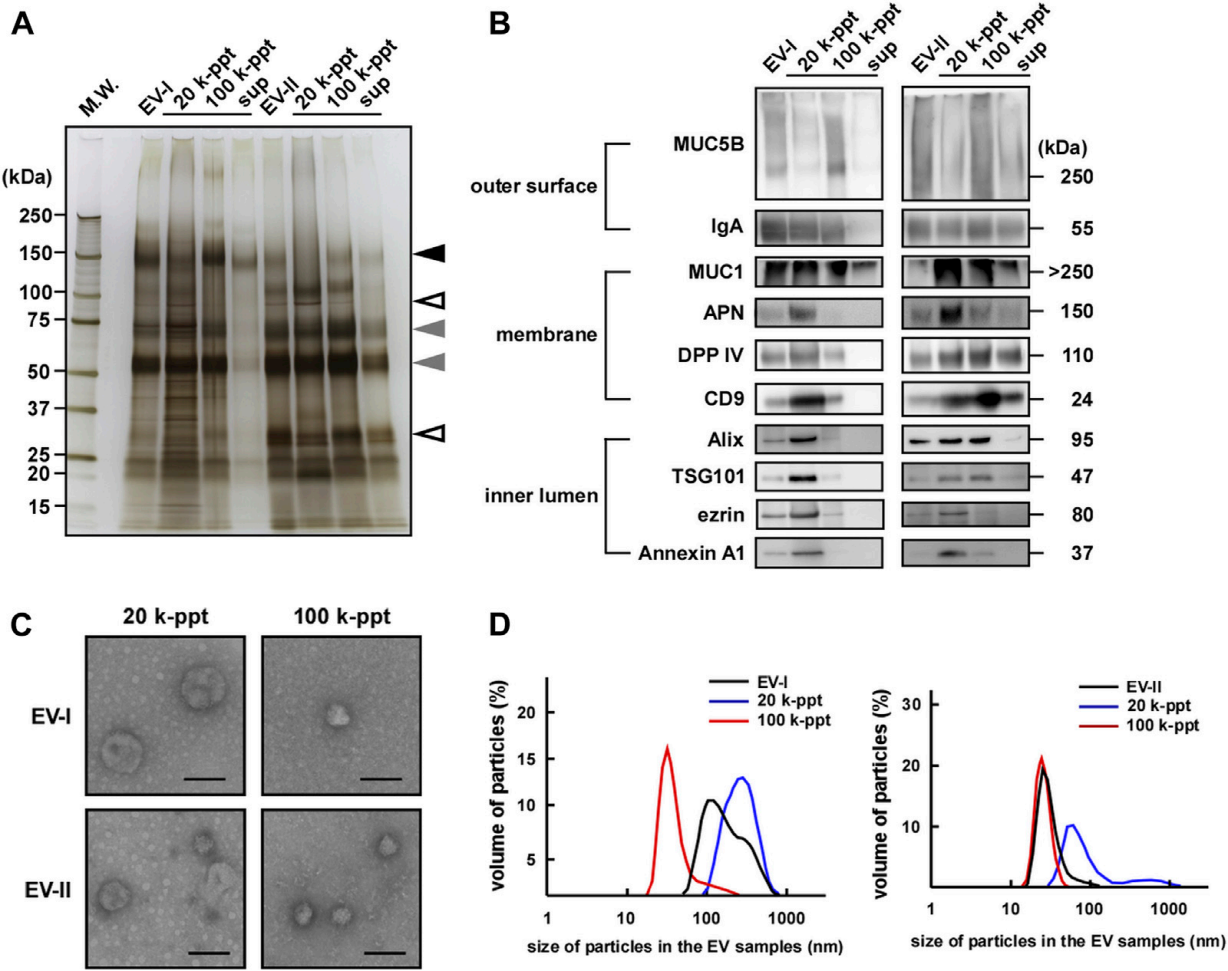
Sequential centrifugation of two types of salivary EVs. Salivary Fr. EV-I and Fr. EV-II were subjected to sequential centrifugation (Figure 1). **(A)** From each fraction, samples of 2 µg of protein were subjected to SDS-PAGE and silver staining (donor A) was performed to visualize the bands. The silver staining of EVs from other donors is shown in Supplementary Figure S2A. Grey arrowheads, protein bands shared by Fr. EV-I and Fr. EV-II; black arrowhead, observed predominantly in Fr. EV-I; open arrowheads, observed predominantly in Fr. EV-II. **(B)** Western blot analysis of proteins located on the outer surface (MUC5B and IgA), membrane (MUC1, APN, DPP IV, and CD9), and inner lumen (Alix, TSG101, ezrin, and Annexin A1) of salivary EVs (donor A). From each EV fraction, samples of 2 µg of protein were subjected to SDS-PAGE, transferred onto PVDF membranes, and immunoblotted with antibodies. Western blots of EVs from other donors are shown in Supplementary Figure S2B. **(C)** Morphological analysis of salivary EV fractions visualized using an electron microscope (donor A). Scale bar, 100 nm. The morphology of EVs from other donors is shown in Supplementary Figure S2D. **(D)** Particle sizes of salivary EVs analyzed via dynamic light scattering (DLS) measurements conducted in triplicate. A typical result is shown (donor A). The mean particle size of EV-I 20 k-ppt was 263 ± 30 nm (7–14 experiments) and that of EV-II 100 k-ppt was 36.5 ± 13 nm (three to six experiments) (Supplementary Figure S2E).

Figure 3A and Supplementary Figure S2A show the protein contents of four fractions separated by size-exclusion and sequential centrifugation procedures. Frs. EV-I and EV-II clearly showed different protein patterns. While both fractions shared ∼70 and ∼55 kDa proteins (grey arrowheads), a ∼150 kDa protein (black arrowhead) was observed predominantly in Fr. EV-I and ∼100 and ∼30 kDa proteins in Fr. EV-II (open arrowheads).

We then compared salivary EV-associated proteins by western blot analysis (Figure 3B; Supplementary Figures S2B,C). In the case of EV-I, the associated protein patterns in the 20 and 100 k-ppt fractions differed: IgA, MUC1, CD9, and DPP IV were detected in both 20 and 100 k-ppt fractions, while APN was little detectable in the 100 k-ppt fraction. Several luminal EV-associated proteins such as Alix, TSG101, ezrin, and Annexin A1 were detected in the 20 k-ppt fraction but barely in the 100 k-ppt fraction. Few proteins were found in the supernatant of Fr. EV-I, although MUC1 was identified.

The 100 k-ppt fraction of EV-II retained EV-associated proteins, although ezrin and Annexin A1 were not evident. Almost all examined EV-associated proteins were detected in 20 k-ppt fractions of EV-II and those samples resembled the 20 k-ppt fractions of EV-I. In addition, the concentrated 20 k-ppt fraction of EV-II contained APN protein despite its lower enzymatic activity (Figure 2; Supplementary Figure S1, and see below). These results suggest that Fr. EV-II was contaminated by Fr. EV-I. The main components of the supernatant fraction of Fr. EV-II, such as MUC1, DPP IV, and CD9, were conceivably cellular debris because luminal EV-associated proteins were not detected.

The morphological features of EVs separated by sequential centrifugation are shown in Figure 3C and Supplementary Figure S2D. Under a TEM microscope, the EV-I 20 k-ppt vesicles were larger in size, whereas vesicles of 100 k-ppt EV-I were smaller. In contrast, the 20 k-ppt fraction of EV-II vesicles showed an intermediate size and the 100 k-ppt fraction of EV-II contained smaller and homogeneous vesicles.

The size distribution measured by DLS confirmed the above-mentioned data obtained by TEM (Figure 3D; Supplementary Figure S2E). The diameters of the EV-I vesicles were distributed heterogeneously between 70 and 1,000 nm, and a shoulder peak was observed. After separation by sequential centrifugation, the diameters of 20 and 100 k-ppt fractions became homogeneous and formed a single peak, being distributed at 100–1,000 and 30–300 nm, respectively. In contrast, the distribution of EV-II vesicle diameters was rather homogeneous, with slight tailing at larger sizes. After separation via sequential centrifugation, the diameters of 20 and 100 k-ppt fractions were homogeneous with a single peak, being distributed at 50–300 and 20–70 nm, respectively. A small proportion of vesicles with larger diameters (>300 nm) were observed in the 20 k-ppt fraction, presumably due to aggregation or fusion during the separation procedure. As shown in Figure 2, Fr. EV-I and EV-II were almost clearly separated, but these fractions appeared to be contaminated with each other. Fractionation by sequential centrifugations resulted in homogeneous fractions of Fr. EV-I and EV-II being obtained as EV-I 20 k-ppt and Fr. EV-II 100 k-ppt, respectively.

Table 1 summarizes the isolation of the two distinctive human salivary EV fractions, EV-I 20 k-ppt and EV-II 100 k-ppt, showing that EV-I 20 k-ppt fraction has APN and DPP IV activities and EV-II 100 k-ppt fraction is rich in DPP IV activity. These findings are consistent with the results of western blot analysis shown in Figure 3B. Taking these findings together, we separated at least two distinctive subpopulations of human salivary EVs with different sizes and protein compositions.

**TABLE 1:** Isolation profiles of salivary EVs from Fr. EV-I or Fr. EV-II by sequential centrifugation.

Sample	Total protein (%)	Total activity of DPP IV (%)	Specific activity of DPP IV (nmol/min/mg protein)	Total activity of APN (%)	Specific activity of APN (nmol/min/mg protein)
Fr. EV-I	100	100	2.00 ± 1.2	100	0.385 ± 0.34
EV-I 20 k-ppt	7.07 ± 1.6	44.8 ± 11	7.41 ± 5.7	40.8 ± 16	3.85 ± 5.7
Fr. EV-II	100	100	8.11 ± 6.1	100	0.162 ± 0.038
EV-II 100 k-ppt	22.6 ± 13	15.8 ± 6.6	5.54 ± 4.4	29.8 ± 12	0.218 ± 0.10

Each salivary EV was subjected to sequential centrifugation (see *Materials and Methods* section). DPP IV activity: Fr. EV-I (*n* = 11), Fr. EV-II (*n* = 10), APN activity: Fr. EV-I (*n* = 8), and Fr. EV-II (*n* = 3). Data are presented as mean ± standard deviation (SD).

### Analyses of EV-I and EV-II vesicle components

To elucidate the characteristic features of the two subpopulations of human salivary EVs, EV-I 20 k-ppt and EV-II 100 k-ppt, of donor A (age 48, female), B, (age 49, male), and J (age 23, female), they were subjected to proteomic analyses. Each sample from three donors was measured in duplicate. The base peak chromatograms of run1 for each donor were shown in Supplementary Figure S3A. Individual differences were observed in the base peak chromatograms. According to the selection criteria described in the “*Materials and Methods*,” total 1,140 proteins were identified in the EV-I 20 k-ppt and EV-II 100 k-ppt fraction (Supplementary Table S3). The results of principal component analysis (PCA) based on all proteomic features of the EV-I 20 k-ppt and EV-II 100 k-ppt fractions are shown in Supplementary Figure S3B. The PCA results show that the EV-I 20 k-ppt fractions segregated from each other, while the EV-II 100 k-ppt fractions integrated, suggesting that the EV-II 100 k-ppt fractions were less different among the three individuals compared with the EV-I 20 k-ppt fractions. A Venn diagram of proteins identified in the EV-I 20 k-ppt and EV-II 100 k-ppt fractions is shown in Supplementary Figure S3C. In total, 1,135 proteins were listed as EV-I 20 k-ppt components, whereas 1,011 proteins were listed as EV-II 100 k-ppt components. A considerable number of proteins were shared by the two sub-populations (1,006). One hundred and twenty-nine proteins in the EV-I 20 k-ppt fraction differed from those in the EV-II 100 k-ppt fraction, whereas the EV-II 100 k-ppt fraction appeared to have a smaller difference (only five proteins) than the EV-I 20 k-ppt fraction. This may reflect differences of biogenesis between them. Characteristic proteins are summarized in Table 2 and are also shown as yellow-highlighted rows in Supplementary Table S3. These proteins were identified in both fractions in this study, and several of them have been known to be present in sEVs or m/lEVs from other sources (Théry et al., 2018). The relative protein abundances between EV-I 20 k-ppt and EV-II 100 k-ppt as determined by the proteomic analyses were similar to the results by Western blotting analyses (Table 2; Figure 3B).

**TABLE 2:** Characteristic proteins in salivary EVs.

No.	Accession	Description	Abundance EV-I 20 k-ppt	Abundance EV-II 100 k-ppt	Sum PEP score	Coverage (%)	MW (kDa)
1	Q9HC84	MUC5B	111.7	88.3	712.4	17	596
6	Q8WUM4	Alix	179.0	21.0	346.7	59	96
7	P15144	APN	155.5	44.5	335.4	36	109.5
12	P15311	Ezrin	198.8	1.2	313.6	54	69.4
23	P01876	Immunoglobulin heavy constant alpha^a^	25.4	174.6	259.4	76	37.6
24	P27487	DPP IV	136.9	63.1	250.3	43	88.2
40	P04083	Annexin A1	197.7	2.3	212.8	63	38.7
59	P15941	MUC1	196.7	3.3	160.8	8	122
120	Q99816	TSG101	182.2	17.8	107.8	41	43.9
771	P21926	CD9	146.6	53.4	13.1	7	25.4

^a^ For IgA, only the identified protein with the highest score is listed. PEP, posterior error probability.

**FIGURE 4:**
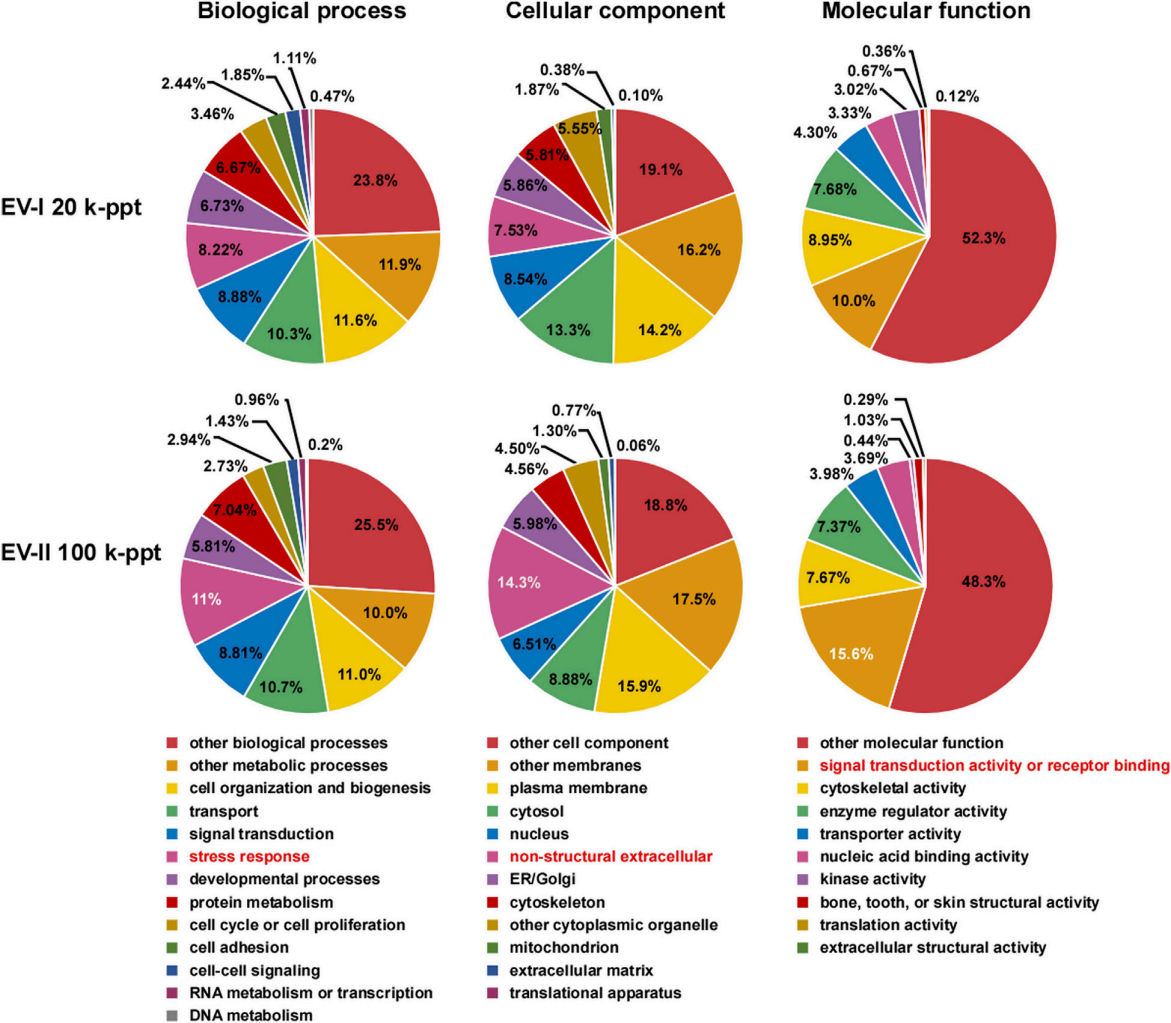
Pie chart of Gene Ontology (GO) analysis of salivary EVs. GO analysis was performed on filtered protein lists of the EV-I or the EV-II fraction, which were confidently identified in all three donors, using Proteome Discoverer 2.4 (Supplementary Table S3). The results are summarized for the three main GO categories: biological processes, cellular components, and molecular functions. Characteristic features are highlighted (red letters).

The proteins in the EV-I 20 k-ppt and EV-II 100 k-ppt fractions were classified into three categories by Gene Ontology (GO) analysis (Figure 4). For the biological process category, EV-II 100 k-ppt was more enriched in proteins involved in the “stress response” than EV-I 20 k-ppt. Furthermore, for the cellular component and molecular function categories, EV-II 100 k-ppt was also more enriched in proteins involved in the “non-structural extracellular” and “signal transduction activity or receptor binding,” respectively, than EV-I 20 k-ppt.

**FIGURE 5:**
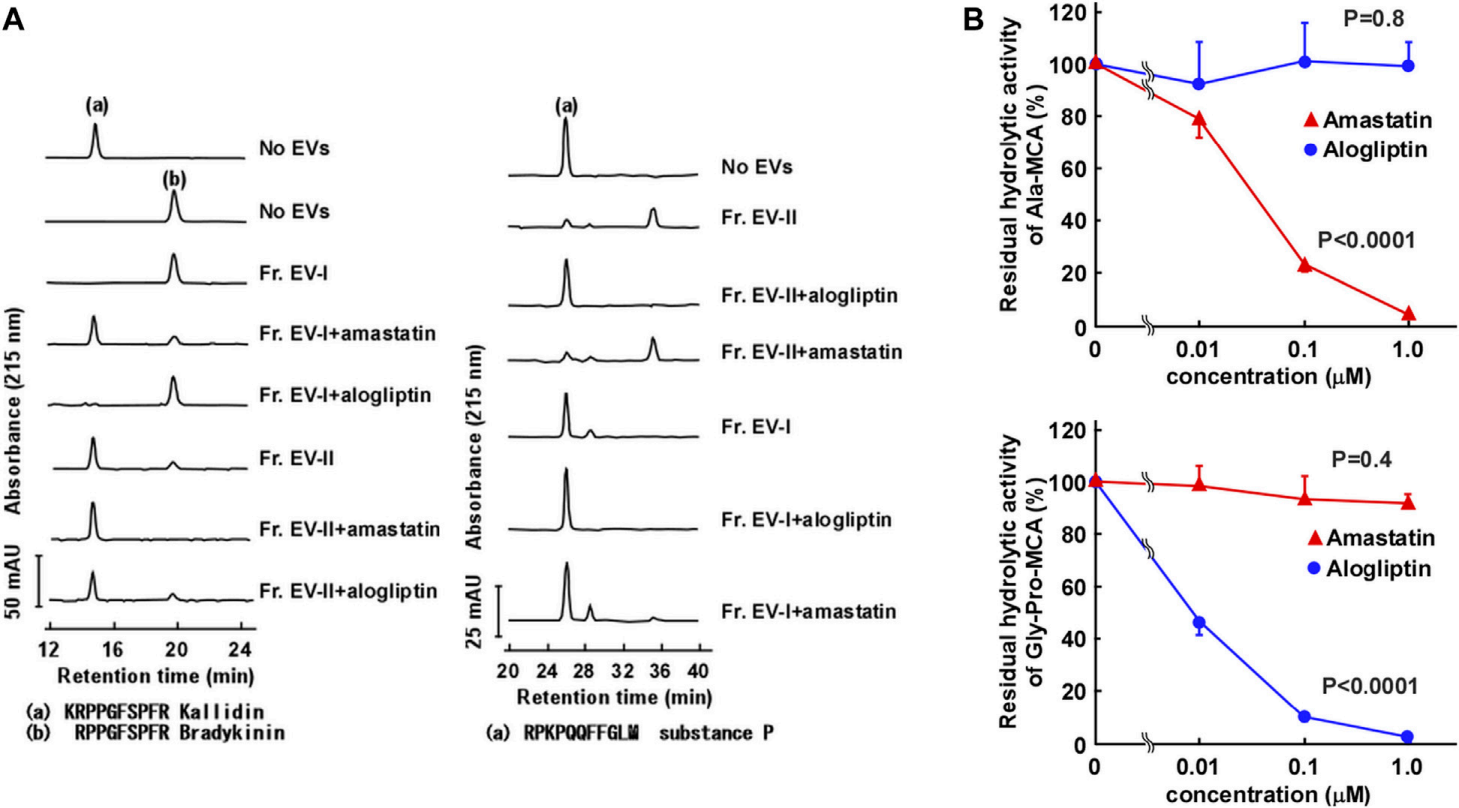
Aminopeptidase activity on the surface of salivary EVs. **(A)** Cleavage of kallidin and substance P by salivary EVs. Kallidin (left) or substance P (right) (25 μM) was incubated with Fr. EV-I or Fr. EV-II (30 μg/mL) in PBS at 37°C for 60 min. To confirm the effect of inhibitors on the aminopeptidase activity, salivary EVs (30 μg/mL) and 1 μM inhibitors (amastatin or alogliptin) were mixed in PBS on ice for 5 min and then incubated with each peptide at 37°C for 60 min. The generated peptides were separated by HPLC. mAU, milli-absorbance units. The cleavage of the peptides by EVs from three donors is shown in Supplementary Table S4. **(B)** Inhibitory effects of Ala-methyl-coumaryl-7-amide (MCA) hydrolytic activity of Fr. EV-I (upper panel) or Gly-Pro-MCA hydrolytic activity of Fr. EV-II (lower panel). Fr. EV-I or EV-II (0.5–2 μg/mL) was preincubated with each inhibitor in PBS on ice for 5 min, and the mixtures were incubated at 37°C for 20 min after the addition of 0.4 mM Ala-MCA for Fr. EV-I or Gly-Pro-MCA for Fr. EV-II. Each bar shows the mean ± standard deviation (SD) (*n* = 3). The residual aminopeptidase activities were compared using one-way analysis of variance (ANOVA).

In order to further characterize the two subpopulations of human salivary EVs, we examined the topology of the membrane proteins such as enzymatic analysis and binding avidity of several antibodies or viral spike proteins. First, we measured peptide-degrading activities of APN and DPP IV (Figure 5A; Supplementary Tables S4, S5). The APN activity was examined by measuring the conversion of Lys-bradykinin (kallidin) to bradykinin (Figure 5A, left). The vesicles in Fr. EV-I converted 84% of kallidin to bradykinin, whereas lower activity was detected in Fr. EV-II (24% conversion). This indicated that Fr. EV-I showed more APN activity than Fr. EV-II, which was consistent with the western blot analyses (Figure 3B). The APN activity of Fr. EV-I was inhibited in a concentration-dependent manner by amastatin, an aminopeptidase inhibitor (Figure 5B, upper panel), confirming that we had measured aminopeptidase activity. Next, the DPP IV activities of Fr. EV-I and Fr. EV-II were compared by measuring the degradation of substance P. Fr. EV-II degraded substance P (81% conversion) more efficiently than Fr. EV-I (20% conversion). In addition, the degradation of substance P by EV-II vesicles was efficiently inhibited by alogliptin, an inhibitor of DPP IV, confirming that the degradation was due to DPP IV (Figure 5A right and 5B lower panels). These results indicate that APN and DPP IV activities are associated with the surface of salivary EVs. The EV-I vesicles were APN-rich and DPP IV-poor, whereas EV-II vesicles were APN-poor and DPP IV-rich, which is consistent with the results shown in the elution profile from the size-exclusion column chromatography (Figure 2).

**FIGURE 6:**
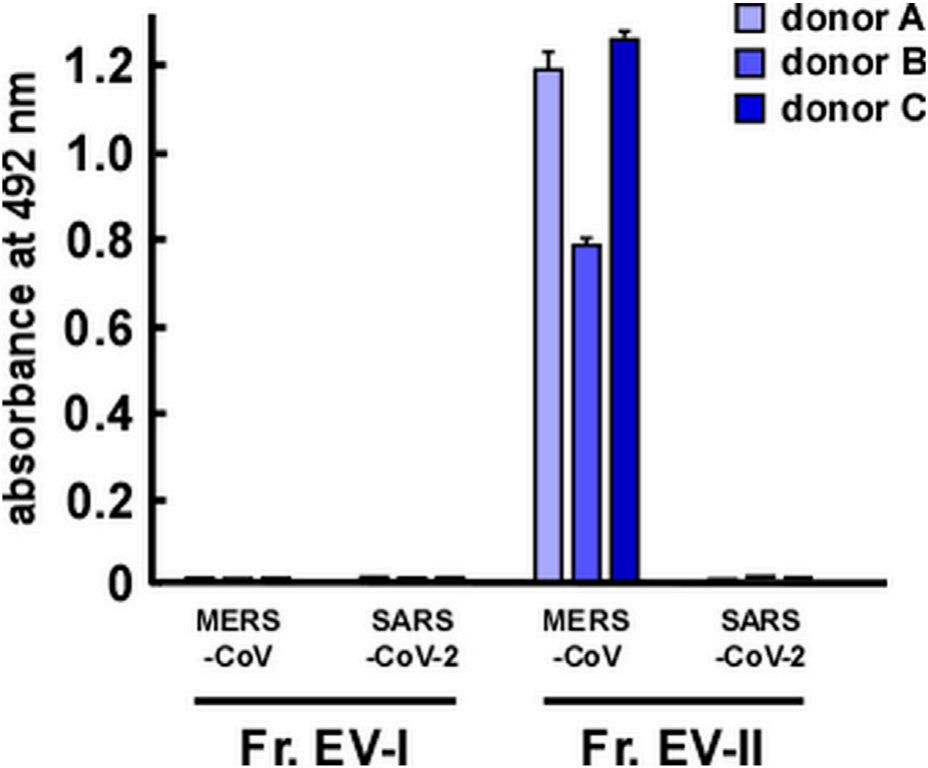
Interaction of salivary EVs and spike proteins of coronaviruses, as determined by enzyme-linked immunosorbent assay. Spike proteins of Middle East respiratory syndrome coronavirus (MERS-CoV) or severe acute respiratory syndrome coronavirus 2 (SARS-CoV-2) were coated onto a 96-well plate. After blocking with bovine serum albumin, Fr. EV-I or Fr. EV-II was added and incubated for 2 h at 25°C. Then, a primary antibody was added and incubated overnight at 4°C. After washing, a secondary antibody was added and reacted at 25°C for 1.5 h. *o*-Phenylenediamine solution was added for detection. After reacting for a certain period (∼30 min), absorbance at 492 nm was measured using a microplate reader.

DPP IV was identified as a receptor of MERS-CoV (Raj et al., 2013), and therefore we investigated whether EVs could bind to spike proteins of coronaviruses. We also examined the binding of EVs to the spike protein of SARS-CoV-2, a receptor that is identified as angiotensin converting enzyme-2 (ACE2) (Zhang et al., 2020). EV-II bound to the MERS-CoV spike protein, but not to that of SARS-CoV-2 (Figure 6). Because ACE2 was not detected as a component of salivary EVs (Supplementary Table S3), EV-II did not bind to the spike protein of SARS-CoV-2, as expected. In contrast, EV-I did not bind to either MERS-CoV or SARS-CoV-2 spike protein, even though it contains DPP IV proteins, as shown in Table 2. This may be because the DPP IV in EV-I was sequestered from the extracellular milieu, and/or DPP IV is not an abundant component in EV-I.

### Membrane environment around EV-associated proteins of EV-I and EV-II vesicles

**FIGURE 7:**
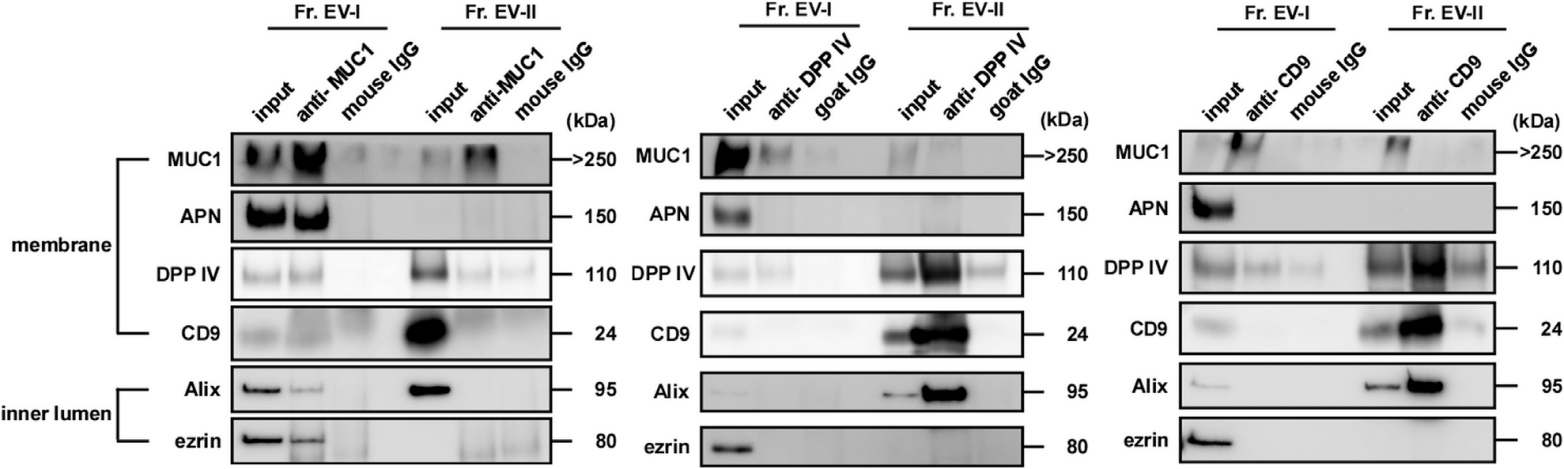
MUC1, DPP IV, and CD9 were expressed on the surface of salivary EVs. Immunoprecipitation of salivary EVs. Salivary EVs (Fr. EV-I or Fr. EV-II) were immunoprecipitated using magnetic beads. Antibody-coupled beads were added to 5 μg of EVs and the mixture was incubated at 4°C for 18 h on a rotator. The proteins eluted from the beads were subjected to SDS-PAGE. Western blots of EVs from other donors are shown in Supplementary Figure S4A. Immunoprecipitation with an anti-APN antibody is shown in Supplementary Figure S4B.

To compare the membrane environment around EV-associated proteins between EV-I and EV-II, immunoprecipitation experiments were performed using antibodies against salivary EV-associated proteins such as MUC1, DPP IV, and CD9 (Figure 7; Supplementary Figure S4). First, the EV fractions were immunoprecipitated with an anti-MUC1 antibody because MUC1 is an integral membrane protein a common component of both EVs. Unexpectedly, the anti-MUC1 antibody failed to co-precipitate the components of Fr. EV-II (i.e., DPP IV, CD9, and Alix), whereas the antibody immunoprecipitated these components of Fr. EV-I (Figure 7, left panel). Considering that MUC1 in Fr. EV-II was precipitated without co-precipitation of CD9, Alix, and DPP IV, MUC1 associated with EV-II vesicles and could be released from the vesicles during the experimental procedure, since MUC1 is a stable heterodimeric complex with an N-terminal subunit and C-terminal subunit, which are linked non-covalently. Alternatively, Fr. EV-II may contain the baseline level of contamination with EV-I.

When the anti-DPP IV antibody was employed, the co-precipitation of several vesicle components along with DPP IV was observed in Fr. EV-II, indicating that these molecules were co-localized in the same vesicles (Figure 7, middle panel). In contrast, co-precipitation of EV-I components by the anti-DPP IV antibody was barely detectable. This is similar to the results observed in binding experiments for viral spike proteins.

Immunoprecipitation experiments were also performed using an anti-CD9 antibody (Figure 7, right panel). The anti-CD9 antibody failed to precipitate itself and the vesicle components of EV-I (Alix and ezrin). In contrast, the antibody immunoprecipitated CD9, Alix, and DPP IV from Fr. EV-II. The amount of CD9 on EV-I was comparable to that on EV-II, as shown in Table 2, suggesting that the antibody could not access its target molecule on EV-I.

We also performed immunoprecipitation using an anti-APN antibody. This antibody worked well in the western blot analyses and precipitated recombinant APN (Supplementary Figure S4B). However, the anti-APN antibody failed to co-precipitate the components of the EV-I vesicles (i.e., MUC1, CD9, Alix, and ezrin). Thus, the anti-APN antibody cannot access its target molecule, which is presumably masked by other membrane components.

**FIGURE 8:**
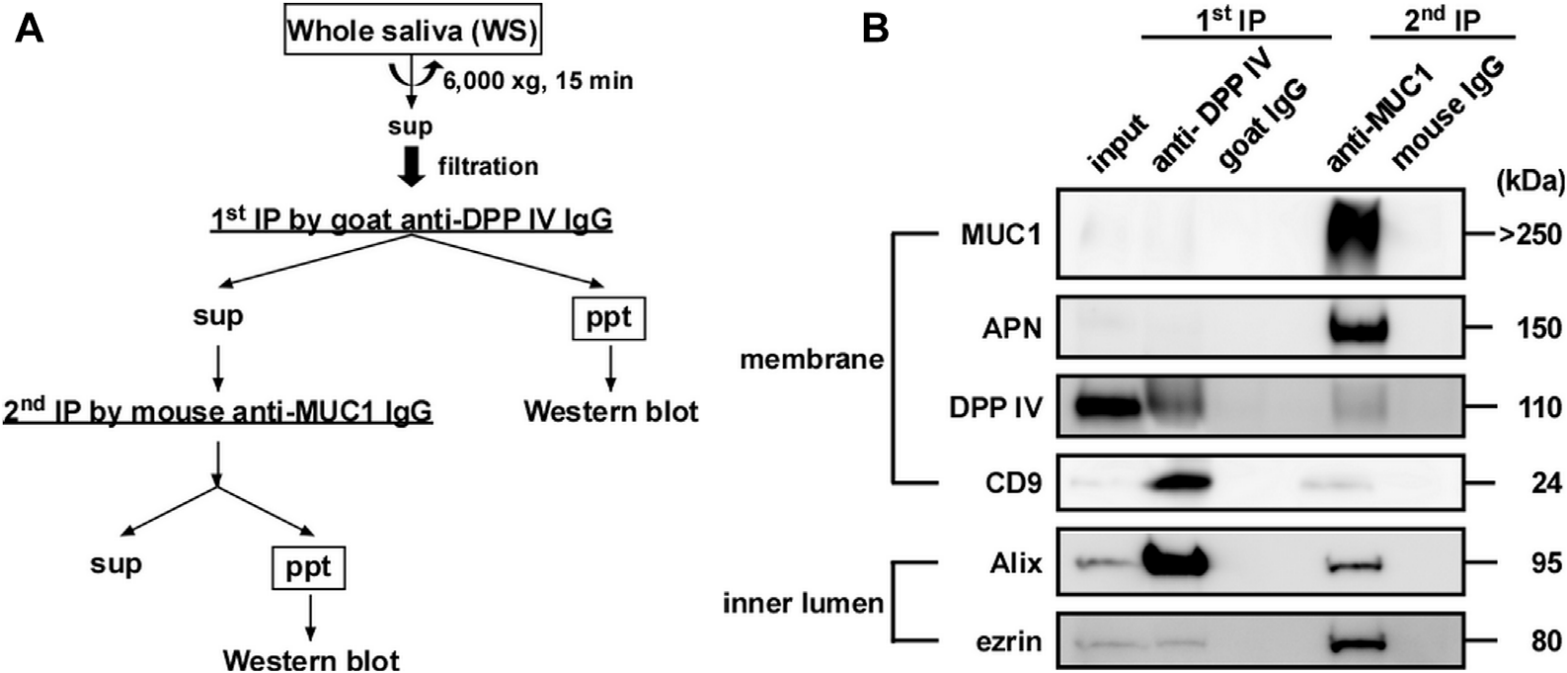
Sequential immunoprecipitation of salivary EVs. **(A)** Flow chart of procedure for sequential immunoprecipitation. Two types of salivary EVs were sequentially immunoprecipitated from the WS using magnetic beads. Pretreated WS (1 mL) was incubated with anti-DPP IV antibody-coupled beads at 4°C for 3 h on a rotator (first immunoprecipitation). After separating the beads, the WS was further incubated with anti-MUC1 antibody-coupled beads (second immunoprecipitation) at 4°C for 18 h. For the control experiment, nonspecific goat IgG-conjugated magnetic beads and nonspecific mouse IgG-conjugated beads were used for the first and second immunoprecipitations, respectively. The immunoprecipitated beads were washed with PBS, and then sample buffer (20 μL) was added and the solution was denatured at 70°C for 10 min. The eluates were then subjected to western blot analysis. **(B)** Western blot analysis of the proteins located on the membrane (MUC1, APN, DPP IV and CD9) and inner lumen (Alix and ezrin) of the salivary EVs (donor A). Western blots of EVs from other donors are shown in Supplementary Figure S5.

On the basis of the results presented in Figure 7, we attempted to construct a simple method to separate EV-I and EV-II using 1 mL of WS via sequential immunoprecipitation, as shown in Figure 8A. The anti-DPP IV antibody was used as the primary antibody to precipitate DPP IV-rich EVs. The resultant supernatant was immunoprecipitated with an anti-MUC1 antibody to precipitate residual MUC1-rich EVs. The first antibody co-precipitated DPP IV, CD9, and Alix, indicating the recovery of EV-II-like vesicles (Figure 8B; Supplementary Figure S5). However, these vesicles lost MUC1, as described above. The second anti-MUC1 antibody immunoprecipitated the EV-I-like vesicles, as shown by co-precipitation with APN, Alix, and ezrin.

## Discussion

In this study, we separated EVs prepared from human WS into two distinct subpopulations by a sequential centrifugation procedure. On the basis of morphological and proteomic analyses, one was large-sized/APN-rich (EV-I) and the other was small-sized/DPP IV-rich (EV-II). DPP IV and APN are aminopeptidases secreted by certain sEVs/exosomes (Kandzija et al., 2019; Gong et al., 2022). DPP IV is thought to play a role in various autoimmune disorders such as inflammatory bowel disease, rheumatoid arthritis, and asthma (Lambeir et al., 2003; Olivares et al., 2018). DPP IV has been shown to induce epithelial cell proliferation and increase fibronectin secretion from cells (Shiobara et al., 2016). We reported that DPP IV on the surface of salivary sEV was stable even under gastric conditions with pepsin (Ogawa et al., 2021). As DPP IV-containing EV-II bound to the spike proteins of MERS-CoV, EV-II in human WS could play a role as a decoy receptor for the virus to modulate the infection.

APN has been associated with different aspects of normal and malignant development (Bauvois and Dauzonne, 2006; Wickström et al., 2011). APN is multifunctional, with various roles such as in enzymatic regulation of peptides, and is involved in certain characteristics of malignant cells, including invasion, differentiation, proliferation, apoptosis, motility, and angiogenesis. Therefore, investigating the correlation between the protein expression levels in salivary EVs and malignant diseases may be interesting.

**FIGURE 9:**
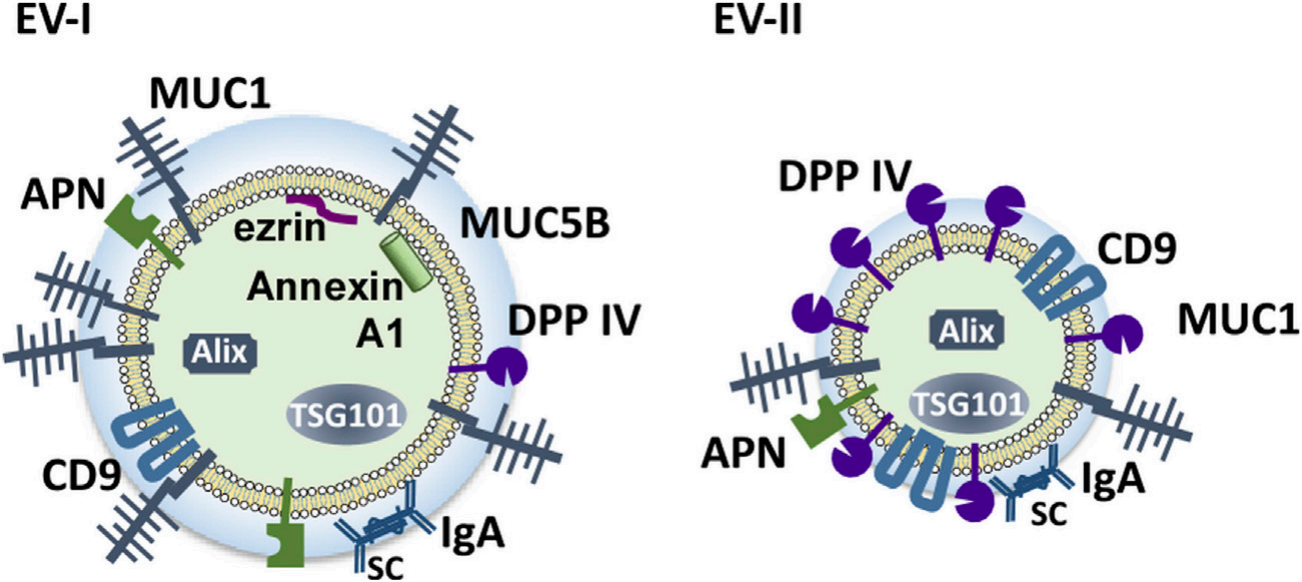
Schematic model of the components of salivary EVs (EV-I and EV-II). Two distinct populations of EVs are present in human WS: large-sized, APN/MUC1-rich, m/l/EVs/microvesicles (EV-I), and small-sized DPP IV/CD9-rich vesicles containing several sEV components (EV-II).

Figure 9 shows schematic structural models of EV-I and EV-II derived from human WS based on this study. EV-I were relatively large vesicles with diameters between 100 and 1,000 nm and contained membrane-bound proteins, including MUC1, CD9, DPP IV, and APN. Characteristically, CD9, DPP IV, and APN were inaccessible to antibodies, suggesting that they are sequestered from the extracellular milieu. Alix, TSG101, ezrin, and Annexin A1 were localized to the vesicles. Since ezrin and Annexin A1 are commonly enriched in m/lEVs/microvesicles (Jeppesen et al., 2019), EV-I should be regarded as m/lEVs/microvesicles. Meanwhile, EV-II vesicles were found to be smaller with diameters between 20 and 70 nm, indicating that they are sEVs. They contained a certain amount of DPP IV, which degraded substance P. Although CD9 was detected as a membrane-bound protein in both EV-I and EV-II vesicles, CD9 in EV-II vesicles, but not in EV-I vesicles, is accessible to the antibody. Moreover, APN of EV-I did not bind to the antibody. These results suggest a difference in the membrane environment around CD9, DPP IV, and APN between EV-I and EV-II. MUC1, a transmembrane mucin, is a large, glycosylated protein with expected molecular weights ranging from 120 to 500 kDa that protrudes out of the cell surface by up to 200–500 nm (Chen et al., 2021). Because EV-I was rich in MUC1, CD9 and APN might be sequestered by MUC1, making them inaccessible to antibodies.

The structural diversity of EVs in terms of their size and protein composition has become increasingly evident. Xiao et al. reported a proteomic analysis of salivary exosomes and microvesicles obtained by filtration and centrifugation (Xiao and Wong, 2012). More recently, Yamamoto et al. also characterized EVs derived from human WS prepared using equilibrium density gradient centrifugation (Yamamoto et al., 2021). They surveyed 111 EV-associated markers and concluded that at least two subclasses were present in saliva. One carried classical exosomal markers, such as CD63 and CD81, and the other was characterized by molecules involved in membrane remodeling or vesicle trafficking, revealing the heterogeneity of human salivary EVs. The same group isolated five types of EVs from the oral fluid of healthy volunteers and patients with oral squamous cell carcinoma using differential centrifugation (Hiraga et al., 2021). The structural and functional significance of the heterogeneity of EVs derived from salivary glands should be further elucidated.

Extracellular proteins in EVs play an important role in cell–cell communication. Hirsova et al. reported that hepatocyte-derived EVs containing tumor necrosis apoptosis-induced ligand (TRAIL), a type II membrane protein, can activate macrophages (Hirsova et al., 2016). In our previous work, we found that endoplasmic reticulum aminopeptidase 1 (ERAP1) was associated with the outer surface of Interferon-gamma-treated but not -untreated murine RAW264.7 cell-derived EVs and enhanced the phagocytic and nitric oxide (NO) synthesis activities of the cells (Goto et al., 2018). In this study, we found that EV-II bound to the MERS-CoV spike protein via DPP IV. DPP IV and IgA were associated with the outer surface of the EV-II 100 k-ppt fraction, suggesting that these vesicles could exert defensive functions.

Although previous studies on EVs mostly focused on sEV/exosomes, recent studies have revealed that m/l/EVs also exhibit important biological activities (Clancy et al., 2021). The simultaneous presence of the two types of salivary EVs found in this study suggests their diverse pathophysiological roles in the oral cavity and gastrointestinal tract, either independently or cooperatively. There is a need for further studies of these roles using saliva-derived EVs from humans with diseases such as asthma. Our method of preparing salivary sEVs (EV-II-like vesicles) and m/l/EVs (EV-I-like vesicles) by sequential immunoprecipitation is anticipated to be efficacious for elucidating the pathophysiological importance of salivary EVs. Moreover, two distinct types of EVs with proteins such as DPP IV and APN are potential tools for the discovery of new markers for diagnosing oral and gastrointestinal diseases and understanding the pathophysiological roles of these EVs.

## Data Availability

The datasets presented in this study can be found in online repositories. The names of the repository/repositories and accession number(s) can be found in the article/Supplementary Material.

## Abbreviations

Alix, ALG-2-interacting protein; BSA, bovine serum albumin; DLS, dynamic light scattering; DPP IV, dipeptidyl peptidase IV; EVs, extracellular vesicles; GO, Gene Ontology; HPLC, high-performance liquid chromatography; MCA, 4-methyl-coumaryl-7-amide; MeCN, acetonitrile; MERS-CoV, Middle East respiratory syndrome coronavirus; MUC1, mucin 1; MUC5B, mucin 5B; PBS, phosphate buffered saline; SARS-CoV-2, severe acute respiratory syndrome coronavirus 2; SD, standard deviation; TFA, trifluoroacetic acid; TEM, transmission electron microscopy; TSG101, tumor susceptibility gene 101; WS, whole saliva.
