# Supplementary material for: Distinguishing two distinct types of salivary extracellular vesicles: a potential tool for understanding their pathophysiological roles
Source: Front Mol Biosci. 2024 Feb 28;11:1278955. doi: 10.3389/fmolb.2024.1278955 (PMC10933032; doi:10.3389/fmolb.2024.1278955)
Supplement: Supplementary file 2 [file DataSheet1.docx]

Supplementary Material

Distinguishing two distinct types of salivary extracellular vesicles: A potential tool for understanding their pathophysiological roles

Yuko Ogawa*, Yuri Miura, Mamoru Ikemoto, Atsushi Ohnishi, Yoshikuni Goto, Kazuma Aoki, Yuki Motokurumada,

Yoshihiro Akimoto, Tamao Endo, Masafumi Tsujimoto, Ryohei Yanoshita

*** Correspondence:** Corresponding Author: y.ogawa@thu.ac.jp

.


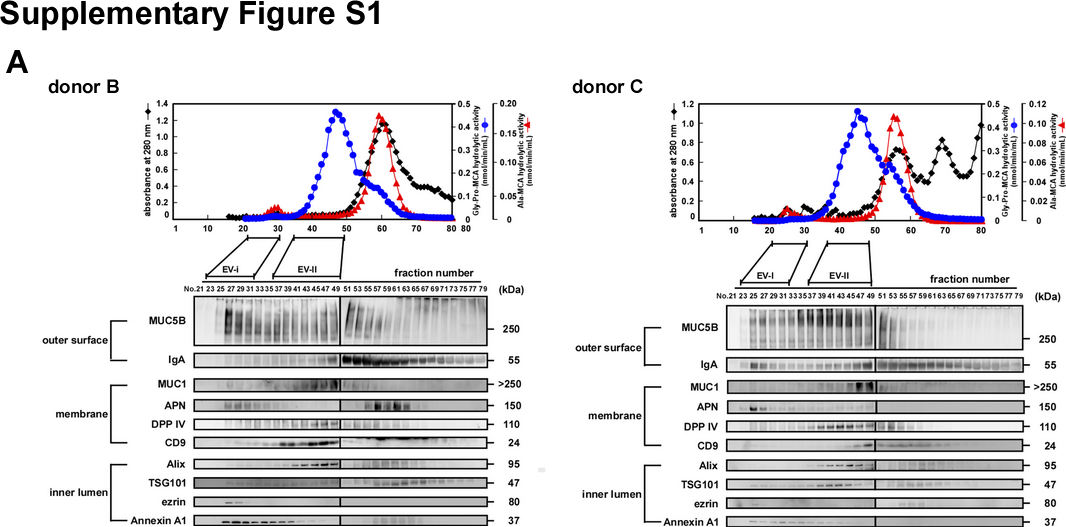


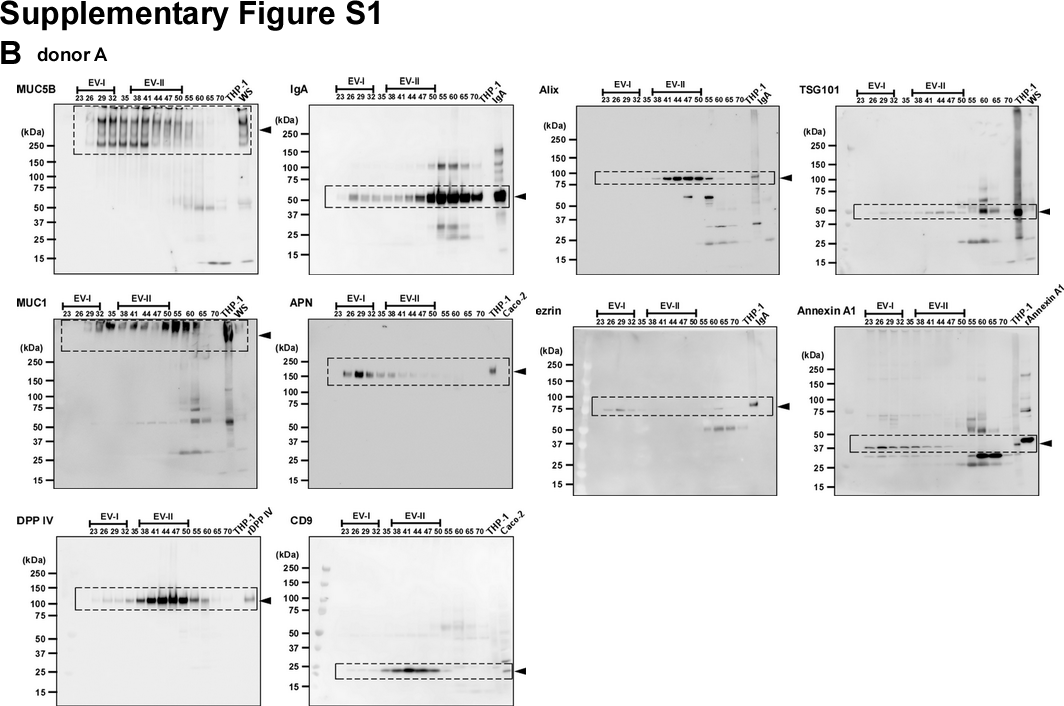


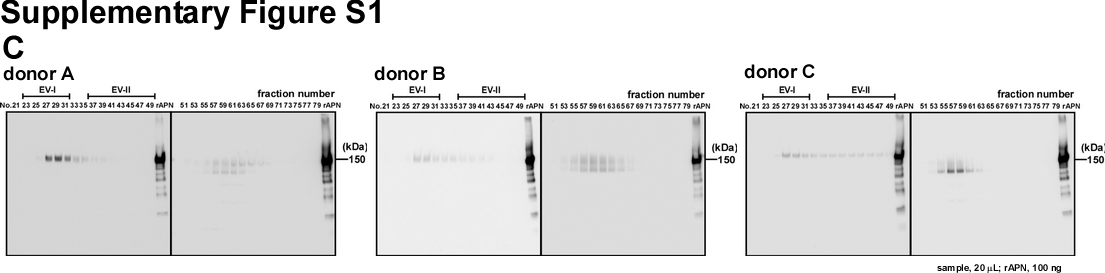


**Supplementary Figure S1. Preparation of extracellular vesicles (EVs) derived from human whole saliva (WS).**

(A) (Upper) Size-exclusion chromatography (Sephacryl S-1000 SF) elution profiles of EVs from fresh human WS (donors B and C). (Lower) Western blot analysis of proteins located on the outer surface (MUC5B and IgA), membrane (MUC1, APN, DPP IV, and CD9), and inner lumen (Alix, TSG101, ezrin, and Annexin A1) of salivary EV fractions eluted from size-exclusion columns (donors B and C). The numbers refer to the different fractions obtained via size-exclusion column chromatography, as shown in the upper panel. Overall, 20 µL of each EV fraction was subjected to sodium dodecyl sulfate–polyacrylamide gel electrophoresis (SDS-PAGE) and analyzed by western blotting. (B) Western blot analysis of size-exclusion chromatography fractions with positive control (donor A). The full range of fractions for size-exclusion chromatography was applied to one gel. Fifteen µL of each EV fraction was subjected to SDS-PAGE and analyzed by western blotting. As positive controls, we used 5~20 µg of cell lysates from THP1 or Caco-2 cell lines, 5 ng of rDPP IV or rAnnexin A1, 1 µg of IgA from human colostrum, or 10 µg of WS. Arrowheads indicate the target protein bands. (C) Detection of APN in size-exclusion chromatography fractions with positive control (rAPN) by western blot analysis. Twenty µL of each EV fraction was subjected to SDS-PAGE. As positive control, 100 ng of recombinant APN (rAPN) was applied to the gels.


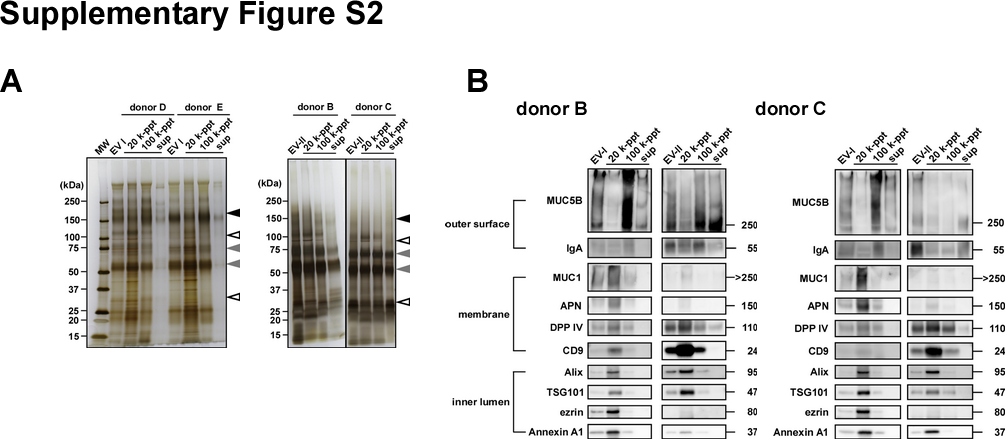


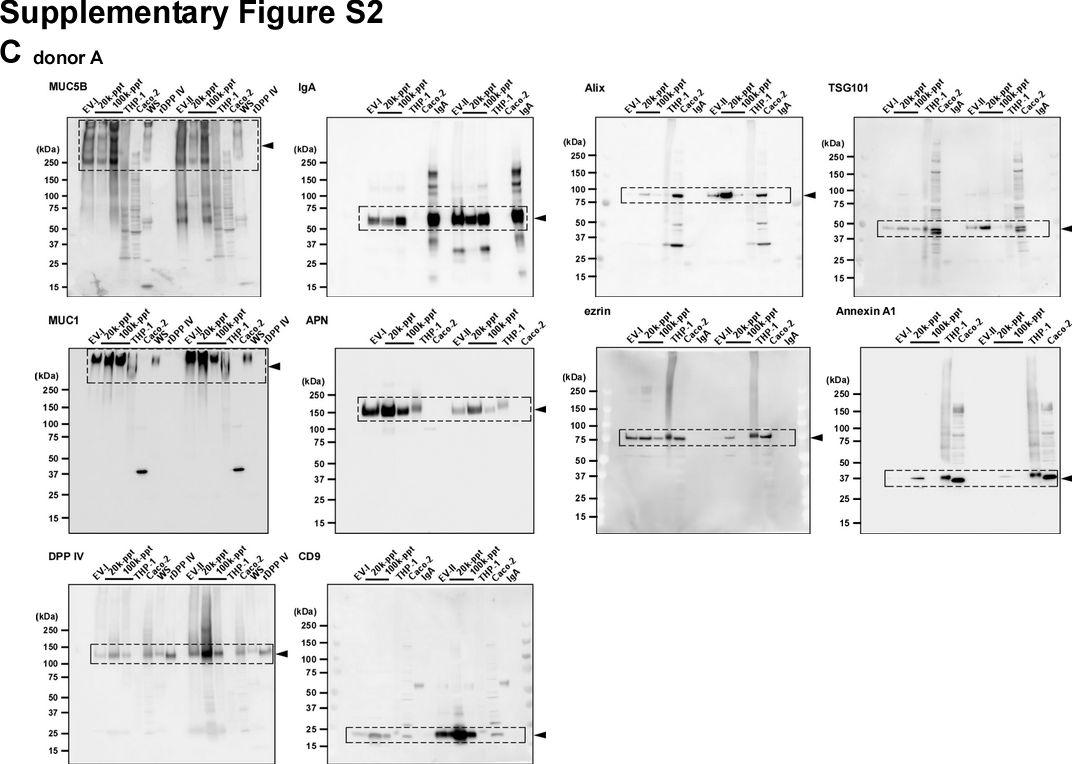


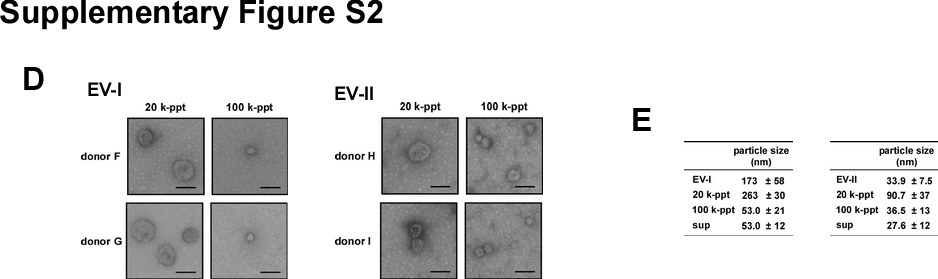


**Supplementary Figure S2. Sequential centrifugation of two types of salivary EVs.**

Salivary Fr. EV-I and Fr. EV-II were subjected to sequential centrifugation. (A) Two micrograms of protein from each fraction were subjected to SDS-PAGE and visualized by silver staining (donors B–E). (B) Western blot analysis of proteins located on the outer surface (MUC5B and IgA), membrane (MUC1, APN, DPP IV, and CD9), and inner lumen (Alix, TSG101, ezrin, and Annexin A1) of salivary EVs (donors B and C). Two micrograms of protein from each EV fraction were subjected to SDS-PAGE, transferred onto polyvinylidene difluoride (PVDF) membranes, and immunoblotted with antibodies. (C) Western blot analysis of EV-I and EV-II with positive controls (donor A). As positive control, we used 5–20 µg of cell lysates from THP-1 or Caco-2 cell line, 5 ng of rDPP IV or rAnnexin A1, 1 µg of IgA from human colostrum, or 10 µg of WS. Arrowheads indicate the target protein bands. (D) Morphological analysis of salivary EV fractions visualized under an electron microscope (donors F–I). Scale bar, 100 nm. (E) The particle size of salivary EVs was analyzed using dynamic light scattering (DLS) measurements, as shown in Figure 3D. Data are shown as the mean ± SD of 7–14 experiments for EV-I and 3–6 experiments for EV-II.


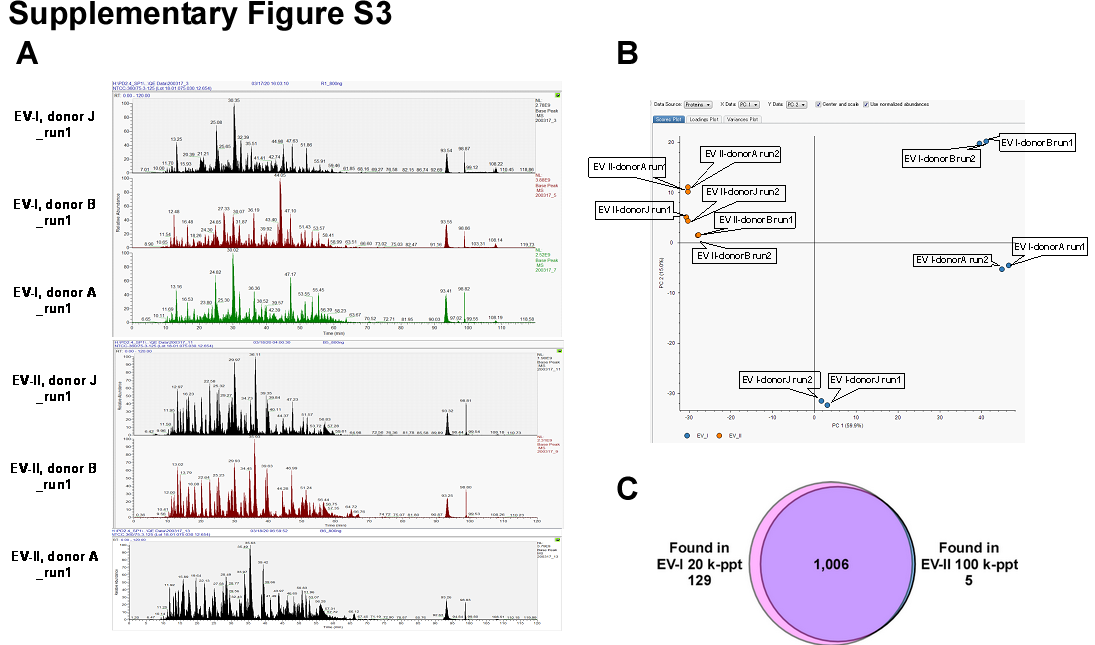
**Supplementary Figure S3. Proteomic analysis of salivary EVs.**

(A) Base peak chromatograms of EV-I 20 k-ppt and EV-II 100 k-ppt fractions from three donors. Measurements were duplicated for each sample, and chromatograms of run1 are shown. (B) Principal component analysis (PCA) based on proteomic features. PCA showed that the EV-I 20 k-ppt fractions segregated from each other, while EV-II 100 k-ppt fractions were integrated, suggesting that EV-II 100 k-ppt was less different among the three individuals than EV-I 20 k-ppt was. (C) Venn diagram of the proteins identified in the EV-I 20 k-ppt and EV-II 100 k-ppt fractions. The numbers indicate the number of proteins identified in EV-I or EV-II.


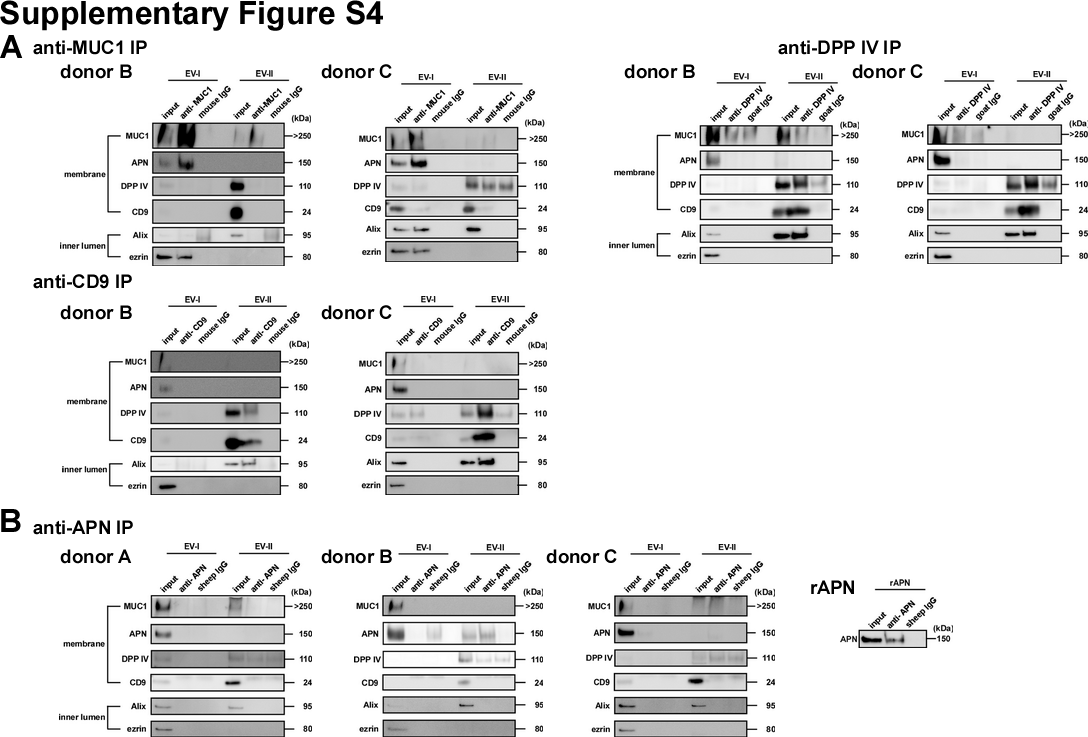


**Supplementary Figure S4. Western blotting of immunoprecipitated proteins derived from EVs of other donors.**

(A) Salivary EVs (donors B and C) were immunoprecipitated using anti-MUC1 antibody-conjugated magnetic beads, anti-CD9 antibody-conjugated magnetic beads, and anti-DPP IV antibody-conjugated magnetic beads. (B) Salivary EVs (donors A–C) were not immunoprecipitated with anti-APN antibody-conjugated magnetic beads, although rAPN (5 ng) was.


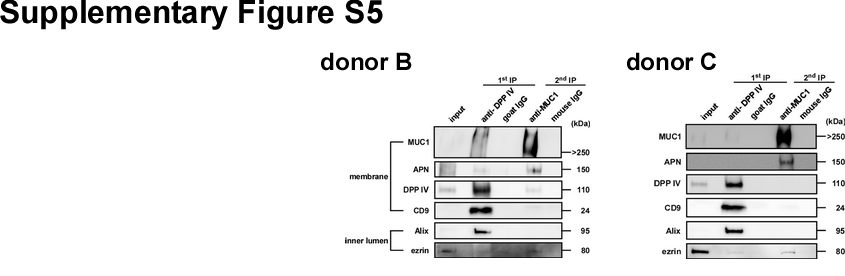


**Supplementary Figure S5. Western blotting of sequential immunoprecipitated proteins derived from the WS of other donors.**

WS (1 mL from donor B or C) was immunoprecipitated using anti-DPP IV antibody-conjugated magnetic beads, and subsequently immunoprecipitated using anti-MUC1 antibody-conjugated magnetic beads.

**Supplementary Table S1 Informations of healthy volunteers**

| **Sample** | **Age** | **Gender** |
| --- | --- | --- |
| **A** | 48 | female |
| **B** | 49 | male |
| **C** | 55 | male |
| **D** | 23 | female |
| **E** | 22 | male |
| **F** | 23 | female |
| **G** | 22 | female |
| **H** | 25 | female |
| **I** | 22 | female |
| **J** | 23 | female |
| **K** | 22 | female |

**Supplementary Table S2 Isolation profiles of salivary EVs from human whole saliva (WS) by size-exclusion chromatography.**

Salivary EVs were isolated from human WS using size-exclusion chromatography (see Materials and Methods).

Protein concentration and DPP IV activity, n = 11; APN activity, n = 9. Data are presented as mean ±SD.

| **Sample** | **Protein concentration in WS (mg/mL)** | **Total protein**  **(%)** |
| --- | --- | --- |
| **WS** | 1.97 ± 0.88 | 100 |
| **Fr. EV-I** | 0.00786 ± 0.010 | 0.360 ± 0.23 |
| **Fr. EV-II** | 0.0278 ± 0.016 | 1.80 ± 0.96 |

| **Sample** | **Total activity of DPP IV**  **in WS (nmol/min/mL)** | **Specific activity of**  **DPP IV**  **(nmol/min/mg protein)** | **Total activity of**  **DPP IV**  **(%)** |
| --- | --- | --- | --- |
| **WS** | 0.626 ± 0.40 | 0.348 ± 0.18 | 100 |
| **Fr. EV-I** | 0.0104 ± 0.0081 | 1.99 ± 1.8 | 1.75 ± 1.5 |
| **Fr. EV-II** | 0.199 ± 0.16 | 8.5 ± 7.0 | 28.5 ± 14 |

| **Sample** | **Total activity of APN in WS (nmol/min/mL)** | **Specific activity of APN**  **(nmol/min/mg protein)** | **Total activity of APN**  **(%)** |
| --- | --- | --- | --- |
| **WS** | 0.00961 ± 0.0046 | 0.0142 ± 0.0080 | 100 |
| **Fr. EV-I** | 0.00108 ± 0.00065 | 0.363 ± 0.34 | 11.0 ± 6.5 |
| **Fr. EV-II** | 0.000700 ± 0.00030 | 0.0577 ± 0.049 | 7.75 ± 2.7 |

**Supplementary Table S4 Cleavage of kallidin and substance P by aminopeptidases of EVs**

Kallidin (A) and substance P (B) (25 μM) were incubated with Fr. EV-I or Fr. EV-II (30 μg/mL) in PBS at 37 °C for 60 min. To confirm the effect of inhibitors on the aminopeptidase activity, salivary EVs (30 μg/mL) and 1 μM inhibitor (amastatin or alogliptin) were mixed in PBS on ice for 5 min and then incubated with each peptide at 37 °C for 60 min. The generated peptides were separated using HPLC. Peak areas of standard peptides were taken as 100%.

**A. Kallidin**

| **Sample** | **Inhibitor** | **Peak area (%)** | | | **Mean** ± **SD** | ***p-value*** |
| --- | --- | --- | --- | --- | --- | --- |
|  |  | **Donor A** | **Donor B** | **Donor C** |  |  |
| **Fr. EV-I** | **none** | 0 | 40.82 | 6.84 | 15.9 ± 22 |  |
|  | **Amastatin** | 72.83 | 92.90 | 67.59 | 77.8 ± 13 | 0.0058 |
|  | **Alogliptin** | 2.69 | 29.81 | 3.73 | 12.1 ± 15 |  |
| **Fr. EV-II** | **none** | 80.18 | 74.49 | 74.13 | 76.3 ± 3.4 |  |
|  | **Amastatin** | 96.26 | 92.40 | 95.22 | 94.6 ± 2.0 | 0.0171 |
|  | **Alogliptin** | 71.05 | 64.37 | 85.77 | 73.7 ± 11 |  |

**B. Substance P**

| **Sample** | **Inhibitor** | **Peak area (%)** | | | **Mean** ± **SD** | ***p-value*** |
| --- | --- | --- | --- | --- | --- | --- |
|  |  | **Donor A** | **Donor B** | **Donor C** |  |  |
| **Fr. EV-I** | **none** | 76.65 | 90.76 | 72.58 | 80.0 ± 9.5 |  |
|  | **Amastatin** | 78.61 | 86.86 | 78.73 | 81.4 ± 4.7 | 0.1 |
|  | **Alogliptin** | 103.33 | 88.89 | 91.21 | 94.5 ± 7.8 |  |
| **Fr. EV-II** | **none** | 14.79 | 34.21 | 8.59 | 19.2 ±13 |  |
|  | **Amastatin** | 12.59 | 22.74 | 7.74 | 14.4 ± 7.7 | 0.0002 |
|  | **Alogliptin** | 81.34 | 78.31 | 79.49 | 79.7 ± 1.5 |  |

**Supplementary Table S5** **Cleavage of kallidin and substance P by the recombinant proteins of aminopeptidases.**

Kallidin (A) and substance P (B) (25 μM) were incubated with a recombinant protein (rAPN or rDPP IV) in PBS at 37 °C for 60 min. The amount of recombinant protein added was adjusted to the total enzymatic activity of Fr. EV-I (for rAPN) or Fr. EV-II (for rDPP IV) derived from three donors (see Materials and Methods). To confirm the effect of inhibitors on the aminopeptidase activity, rAPN or rDPP IV and 1 μM inhibitor (amastatin or alogliptin) were mixed in PBS on ice for 5 min and then incubated with each peptide at 37 °C for 60 min. The generated peptides were separated using HPLC. All measurements were performed in triplicate.

**A. Kallidin**

| **Sample** | **Inhibitors** | **Peak area (%)** | | | **Mean** ± **SD** | ***p-value*** |
| --- | --- | --- | --- | --- | --- | --- |
|  |  | **1** | **2** | **3** |  |  |
| **rAPN** | **none** | 48.4 | 44.0 | 41.2 | 44.5 ± 3.6 |  |
|  | **Amastatin** | 103.2 | 94.6 | 87.7 | 95.2 ± 7.8 | *p* < 0.0001 |
|  | **Alogliptin** | 53.3 | 51.3 | 48.6 | 51.1 ± 2.4 |  |
| **rDPP IV** | **none** | 95.5 | 101.4 | 96.4 | 97.8 ± 2.6 |  |

**B. Substance P**

| **Sample** | **Inhibitors** | **Peak area (%)** | | | **Mean** ± **SD** | ***p-value*** |
| --- | --- | --- | --- | --- | --- | --- |
|  |  | **1** | **2** | **3** |  |  |
| **rAPN** | **none** | 92.8 | 98.7 | 100.1 | 97.2±1.4 |  |
| **rDPP IV** | **none** | 30.9 | 37.3 | 35.04 | 34.4±3.2 |  |
|  | **Amastatin** | 35.9 | 31.5 | 28.9 | 32.1± 3.6 |  |
|  | **Alogliptin** | 88.0 | 90.1 | 98.7 | 92.3 ± 5.6 | *p* < 0.0001 |
